# Why a landscape view is important: nearby urban and agricultural land affects bird abundances in protected areas

**DOI:** 10.7717/peerj.10719

**Published:** 2021-07-28

**Authors:** Gregory Duncan Duckworth, Res Altwegg

**Affiliations:** 1 Statistics in Ecology, Environment and Conservation, Department of Statistical Sciences, University of Cape Town, Cape Town, South Africa; 2 African Climate and Development Initiative, University of Cape Town, Cape Town, South Africa

**Keywords:** Protected areas, Land-use change, Population ecology, Atlas data, Population modelling, Landscape ecology, Conservation, Avian ecology

## Abstract

Protected areas are one of the primary conservation tools used worldwide. However, they are often embedded in a landscape that is intensely used by people, such as for agriculture or urban development. The proximity of these land-use types to protected areas can potentially affect the ecological effectiveness (or conservation effectiveness) of protected areas. In this article, we examine to what degree adjacent agricultural and urban land uses affect the ecological effectiveness of protected areas over the greater Gauteng region of South Africa. We selected 198 common, resident bird species, and analysed detection/non-detection data for these species collected over regular grid cells (approximately 61 km^2^ in area). For each species, we estimated abundance per grid cell with the Royle-Nichols model in relation to the proportion of protected area as a covariate. Our study focused on how this relationship between proportion of protected area and abundance (which we term the ‘protection–abundance relationship’) changed as a function of other land-use types in the grid cell. Specifically, we examined the interaction effects between protected area and both urban and agricultural land-use type per grid cell on bird abundance. We assigned each species to one of seven guilds, namely: frugivores, gleaners, granivores, ground-feeders, hawkers, predators and vegivores, and examined how the protection–abundance relationship varied across guilds in relation to agriculture and urban area. As urban area within a grid cell increased, the protection–abundance relationship became more positive for 58% of all species. At the level of guilds, the protection–abundance relationship became more positive for two guilds (granivores and ground-feeders), more negative for frugivores, and remained unchanged for the other four guilds (gleaners, hawkers, predators and vegivores). As agricultural area within a grid cell increased, the protection–abundance relationship became more positive for 49% of all species. At the guild level, the protection–abundance relationship became more positive for six guilds (frugivores, gleaners, ground-feeders, hawkers, predators and vegivores) and remained unchanged for the granivores. Our results show land-use type near protected areas modified the effect protected areas had on bird abundances, and hence the ecological effectiveness of protected areas. Our results suggest that protected areas should be viewed as constituents within the landscape, rather than islands of protection.

## Introduction

Protected areas are one of the key strategies worldwide for conserving the earth’s natural habitat and biodiversity (Gaston et al., 2008; James, Gaston & Balmford, 1999; Parrish, Braun & Unnasch, 2003). Although not a sole solution to conservation challenges, protected areas are globally considered effective at conserving biodiversity (Chape et al., 2005; Gaston et al., 2006). Every year, substantial amounts of financial and human resources are allocated to maintain current protected areas, and develop new ones (Bruner, Gullison & Balmford, 2004; James, Gaston & Balmford, 1999; Naidoo et al., 2006; Rands et al., 2010). Protected areas are generally designed by conservation managers to conserve biodiversity, habitat, and to promote ecosystem functionality and services such as pollination and water provision (Gaston et al., 2008). In the last few decades these goals have broadened to include social aspects, such as national development and poverty reduction (Naughton-Treves, Holland & Brandon, 2005).

In general, protected areas are expected to confer a net positive effect at conserving biodiversity and habitat (Gaston et al., 2008). However, a large body of literature shows that many protected areas are failing to conserve the flagship species they were intended to conserve (Brashares, Arcese & Sam, 2001; Cantú-Salazar et al., 2013; Craigie et al., 2010; Newmark, 1996; Ogutu et al., 2011; Rands et al., 2010; Rodrigues et al., 2004; Western & Henry, 1979). Furthermore, biodiversity in general is declining in some protected areas (Craigie et al., 2010; Hoekstra et al., 2002; Watson et al., 2014). Consequently, despite the large allocation of time and financial resources invested in protected areas (Gaston et al., 2008; Hockings et al., 2006; Rodrigues et al., 2004), there is growing concern by scientists and conservationist that protected areas are not achieving the conservation goals set out for them (Brashares, Arcese & Sam, 2001; Hilton-Taylor et al., 2004; Newmark, 1996).

One major reason that the conservation goals set out for protected areas may not be achieved could be due to land-use types neighbouring protected areas, and in particular, urban and agricultural area (DeFries et al., 2007; Hansen & Defries, 2007; Leroux & Kerr, 2013). For example, the intensity of human settlements situated within or around a protected area is strongly positively correlated with biodiversity declines, species extinction, fire frequency, poaching, and general habitat degradation within (or along the borders of) protected areas (Brashares, Arcese & Sam, 2001; Cardillo et al., 2004; Herremans & Herremans-Tonnoeyr, 2001; Knapp et al., 2008; Parks & Harcourt, 2002). Additionally, the density of roads and other infrastructure correlates highly with biodiversity loss within and outside protected areas (Trollope, White & Cooke, 2009). It appears that in general, people preferentially settle near protected areas; urban settlements are located outside or near protected areas at a higher rate than is expected by chance (Chown et al., 2003), and the population growth rate of human settlements just outside protected areas was almost double that of their rural counterparts for 306 protected areas within 45 Latin American and African countries (Wittemyer et al., 2008). Other studies report similar findings elsewhere in the world (Luck, 2007). It is therefore important to understand how the capacity of protected areas to conserve biodiversity and habitats (i.e. the ecological effectiveness of protected areas) is affected by adjacent non-natural land types (e.g. urban areas).

The negative impacts of agriculture on biodiversity have been widely acknowledged and reported (Bengtsson, Ahnstrom & Weibull, 2005; Kleijn et al., 2001; Tscharntke et al., 2005). Activities associated with agricultural practices such as drainage, tillage, run-off, and fertilizing are harmful to biodiversity, and therefore, biodiversity in agricultural areas is often reported to be lower than in protected areas (Darkoh, 2003; Feehan, Gillmor & Culleton, 2005). Furthermore, intensive farming can have negative long-term effects on biodiversity beyond the area that is actually farmed (Stoate et al., 2001, 2009). Consistent large-scale agricultural practices can decrease the quality of the soil, air, and water within entire landscapes, and consequently alter the shape, structure and composition of the landscape (Billeter et al., 2008; Stoate et al., 2001). Rapid changes in landscape structure compromise important ecosystem processes such as pollination (Kremen & Ricketts, 2000; Potts et al., 2010), nutrient recycling (Alberola et al., 2008; Goulding, Jarvis & Whitmore, 2008; Pollock et al., 2008), and water purification (Garnett et al., 2013; Pretty, 2008). Because protected areas are imbedded within landscapes of multiple uses, including agriculture, they can be subjected to cascading negative effects of large-scale agricultural practices, which may, in turn, negatively affect their ecological effectiveness.

Multiple studies have focussed on the effects of the surrounding landscape on protected areas (Chazdon et al., 2009; Craighead, 1978; DeFries et al., 2007; DeFries, Karanth & Pareeth, 2010; Greve et al., 2011; Leroux & Kerr, 2013; Turner, Lambin & Reenberg, 2008). However, relatively few studies have explicitly studied how land-use types adjacent to, or near protected areas affect the ecological effectiveness of protected areas. We examined patterns of abundance of common non-migratory bird species in relation to land use surrounding protected areas. Abundance is a good measure for the ecological status of species, as it is used as a measure of extinction risk (IUCN, 2000; Gaston, 2010). Birds are good environmental indicators of ecosystem health, easy to observe, and well monitored, making them an ideal choice for this type of study (Furness & Greenwood, 1993; Greenwood, 2004). Common bird species have been shown to be important drivers of ecosystem patterns, and functions, such as primary productivity and nutrient cycling (Lennon et al., 2011; Winfree et al., 2015). A decline in abundances and diversity of common species can indicate drastic declines in ecosystem integrity (Gaston, 2011). Monitoring abundances of common birds within protected areas therefore gives a good representation of the ecological integrity of protected areas, and consequently, their ecological effectiveness.

We used data collected from regular grid cells across the greater Gauteng area in South Africa to estimate how the abundance of common, resident bird species varied as the proportion of protected area within a grid cell increased. We statistically examined the effect of the amount of protected area on species’ abundances within grid cells, treating the proportion of each grid cell that was protected as a covariate in a modelling framework that accounts for imperfect detection. We refer to the relationship between the proportion of protected area and abundance as the protection–abundance relationship, and use it as a measure of ecological effectiveness of protected areas. A positive protection–abundance relationship indicated that abundance increased as the proportion of protected area within a landscape increased; from this, we infer that protected areas were ecologically effective for that species. Conversely, a negative protection–abundance relationship indicated the opposite. Here, we examine how the protection–abundance relationship changes with increasing proportion of urban and agricultural area in the same grid cell. The ecological focus of this study was to determine the way in which the protection–abundance relationship varied with increases in either urban or agricultural land-use types, rather than the protection–abundance relationship itself (see Duckworth & Altwegg, 2018).

This study addressed two key aims; (1) for what percentage of species does the protection–abundance relationship increase or decrease with increasing proportions of urban and agricultural area in the same grid cell? (2) What is the average change in magnitude of the protection–abundance relationship with increasing proportions of urban and agricultural area in each grid cell. We expected a high degree of variation in the way the protection–abundance relationship changed in response to increases in agricultural and urban area near protected areas. For example, insectivorous species can be sensitive to non-natural habitats such as urban and agricultural land-use types, and tend to be less diverse and abundant in those areas (Canaday, 1996; Sekercioglu et al., 2002; Watson, 2015). Furthermore, ground-feeding and hawking insectivorous birds tend to be more abundant inside protected areas than outside them, on average (Duckworth & Altwegg, 2018). Thus, for insectivorous guilds, we expect the average protection–abundance relationship to become more positive (more steep) as the proportion of agricultural or urban area near protected area increases. Conversely, granivores (species that primarily feed on grains and seeds) may benefit from both urban and agricultural areas, and have shown to persist well in both these land-use types (Chace & Walsh, 2006; Duckworth & Altwegg, 2018; Sekercioglu, 2012; Whittingham & Markland, 2002). Thus, we hypothesise that the average protection–abundance relationship for granivores will decrease as the proportion of both agricultural and urban land outside protected areas increases, as granivores persist preferentially in agricultural and urban areas. On the other hand, raptor species in southern Africa have been shown to respond negatively (by decreasing in abundance and range extent) to human-modified landscapes (Brandl, Utschick & Schmidtke, 1985; Herremans & Herremans-Tonnoeyr, 2000), in particular, to agricultural areas where they are actively persecuted by farmers (Anderson, 2000; Boshoff, 1980). Thus, we expect the average protection–abundance relationship for predators to become significantly more positive as the proportion of both agricultural and urban areas outside protected areas increases.

## Methods

### Study area

**Figure 1:**
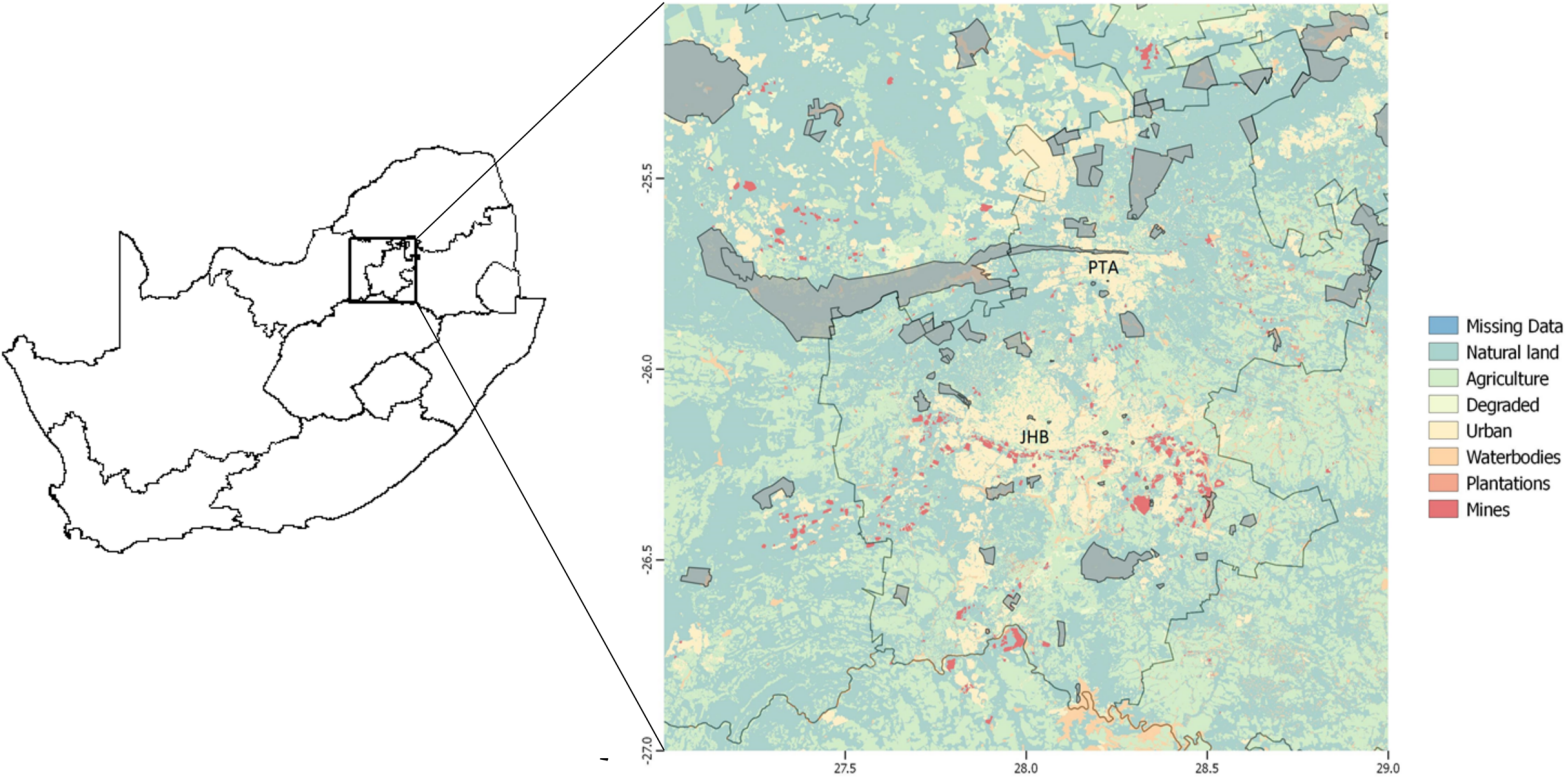
Study area. Land-use types over the greater Gauteng area, South Africa, which constituted the study area, and included major metropolitan areas Johannesburg (JHB) and Pretoria (PTA). Thicker black lines indicate provincial boundaries and Gauteng province is the province in which Johannesburg and Pretoria are situated. Protected areas are indicated in grey.

Our study area consisted of a heterogeneous landscape (a square with coordinates at the NW corner: 25S 27E and SE corner: 27S 29E) that included the greater Gauteng Province of South Africa. It consisted of a rich mix of urban and other heavily human-modified land-use types, as well as protected areas, and the study area included the cities of Pretoria and Johannesburg (Fig. 1), which are two of the most densely populated cities in South Africa (Statistics South Africa, 2012).

The study area was approximately 35,000 km^2^, and comprised of eight land-use types (Fig. 1): mines (0.80% of total land use); plantations (0.32%); waterbodies (2.80%); degraded (2.54%); protected area (6.40%); urban (8.13%); agriculture (28.71%); and natural land (50.30%). Here, natural land refers to land that is not primarily used for any of the other aforementioned land uses. Therefore, in addition to representing the naturally occurring vegetation (grass and trees), it can also represent small holdings, open plots alongside roads or between agricultural area, and recreational land uses (such as sports-fields, parks and lawns). Land-use data were provided by the South African National Biodiversity Institute (South African National Biodiversity Institute (SANBI), 2009) at a 30 m × 30 m resolution.

Urban, agricultural and natural land made up approximately 87% of the land-use cover over the study area, and are known to be influential in affecting bird distributions (Brandon, Redford & Sanderson, 1998; Knapp et al., 2008; Santos et al., 2008; Zhang et al., 2011). We therefore exclusively examined these land-use types in addition to protected area in this analysis.

### Species detection/non-detection data

We used bird detection data from the second Southern African Bird Atlas Project (SABAP 2) which started in June 2007 (Harebottle et al., 2007) and was on-going in 2018, when we performed the analysis. SABAP 2 is a citizen science project whereby registered volunteers submit checklists of birds they observed during a fixed time period within a pre-defined area called a pentad, which is 5′ × 5′ in dimension (unit is arcminutes; approximately 61 km^2^ in area). Volunteers must have spent at least 2 hours, but not more than five days searching for birds within each pentad. Only the presence of bird species is recorded per pentad, not the number of birds seen. Observers were asked to sample all habitats within the pentad. Unusual records were scrutinized by a vetting committee, who either accepted or rejected the record based on supporting information (Harebottle et al., 2007). Every checklist constitutes a detection (if the species was recorded) or non-detection (if the species was not recorded) for all species that occur in the area. We treat each checklist as an independent survey to a particular grid cell.

We considered only common, resident bird species within the study area, and omitted any nomadic, alien, and migratory species, totalling 198 species. We included bird atlas data that were collected and submitted to the project between the beginning of January 2014 and end of December 2015. The years 2014 and 2015 had enough data to support robust data analyses, whilst being short enough a time period to assume abundance did not change markedly in this period (see “Discussion”). Because pentads within the study area were not surveyed the same number of times, like Broms et al. (2014), we randomly selected 100 checklists for pentads that had more than 100 checklists (Fig. A1, Appendix). The study area covers 576 pentads (a 24 pentad by 24 pentad grid), for which 10,400 checklists were submitted at an average of approximately 18 checklists per pentad (min. 1 and max. 468)

Each species was assigned to a guild based on information in Hockey, Dean & Ryan (2005). The definition of a guild is based on a species’ primary food source, and its primary foraging mode. We distinguished between seven guilds, namely: frugivores (species that primarily consume fleshy fruit, totalling nine species); gleaners (species that primarily consume insects and other invertebrates caught off plants, totalling 30 species); granivores (species that primarily consume seeds and grains, totalling 48 species); ground-feeders (species that primarily consume insects and invertebrates caught off the ground, totalling 62 species); hawkers (species that primarily consume insects and other invertebrates caught in the air, totalling 11 species); predators (birds of prey, species that primarily consume the flesh of vertebrates, totalling 19 species), and vegivores (vegetative herbivores; species that primarily consume vegetative parts of plants, totalling 19 species). Table A2 (Appendix) shows the species that constitute each guild. In total, we considered 198 species.

### Analyses

To model the abundance of each species per pentad, we used an extension of traditional occupancy models, known as the Royle-Nichols model of abundance (Royle & Nichols, 2003). Briefly, occupancy models are a class of models which use detection/non-detection data to estimate the probability that a species occurs within a specified area (a pentad in this case). The Royle-Nichols model infers abundance based on detection/non-detection data. These models account for the fact that most species are not observed perfectly in each habitat in which they occur (MacKenzie & Kendall, 2002; Pellet & Schmidt, 2005). Failure to account for non-detection may bias parameter estimates (Boulinier et al., 1998; MacKenzie et al., 2006; Nichols et al., 1998).

We tested for collinearity among the covariates using the Variance Inflation Factor. The Variance Inflation Factors for all our covariates were below 5 (Table A1, Appendix), suggesting multicollinearity wasn’t a problem in our dataset (Kock & Lynn, 2012; Montgomery & Peck, 1992).

#### Abundance models

The Royle & Nichols (2003) model exploits the relationship between the latent abundance at pentad }{}i\ \left({{N_i}} \right), the probability of detecting the species at pentad }{}i during survey }{}j\ \left( {{p_{ij}}} \right), and the probability of detecting an individual \left( {{r_{ij}}} \right) by:

(1) }{}{p_{ij}} = 1 - {\left( {1 - {r_{ij}}} \right)^{{N_i}}}

where, at pentad }{}i and survey }{}j, }{}{N_i} is the latent abundance, }{}{r_{ij}} is the detection probability for an individual, and }{}{p_{ij}} is the pentad-specific detection probability.

The detection of an individual during survey }{}j at pentad }{}i is modelled using a binomial distribution:

(2) }{}{w_{ij}}\sim Binomial\left( {{r_{ij}}} \right)

We modelled the individual detection probability }{}{r_{ij}} with survey specific covariates using a logit link function in the form:

(3) }{}logit\left( {{r_{ij}}} \right) = {\alpha _0} + {\alpha _1} \times {h_{ij}}

where }{}{h_{ij}} is the logarithm of the number of hours spent birding during survey }{}j at pentad }{}i, and the \alpha are coefficients to be estimated by the model.

The latent abundance across pentads, }{}{N_i}, was modelled using a Poisson distribution with rate parameter \lambda:

(4) }{}{N_i}\sim Poisson\left( {{\lambda _i}} \right)

and \lambda was modelled with pentad specific covariates using the log link function:

(5) \eqalignb{log\left( {{\lambda _i}} \right) =\,& {{\beta}_0} + {{\beta}_1} \times P{A_i} + {{\beta}_2} \times Urba{n_i} + {{\beta}_3} \times Agri{c_i} + {{\beta}_4} \times P{A_i} \times Urba{n_i} +\cr & {{\beta}_5} \times P{A_i} \times Agri{c_i} + {{\beta}_6} \times Savann{a_i}}

where the proportion of pentad }{}i occupied by protected area, urban area, agriculture area, and savanna vegetation is represented by }{}P{A_i}, }{}Urba{n_i}, }{}Agri{c_i}, and }{}Savann{a_i} respectively, and the \beta are the coefficients to be estimated by the model. Table 1 gives a summary of each model beta and its ecological interpretation.

**Table 1:** Summary of model coefficients. Summary of the ecological interpretations of the beta coefficients specified in Eq. 5.

Model Beta (β)	Name	Interpretation (across each pentad)
β_0_	Intercept	Average bird abundance in natural land (and zero proportion of PAs, urban, or agricultural in pentad.)
β_1_	PAs	Measures how average bird abundance varies as the proportion of protected areas increase (protection-abundance relationship.)
β_2_	Urban	Measures how average bird abundance varies as the proportion of urban areas increase.
β_3_	Agricultural	Measures how average bird abundance varies as the proportion of agricultural areas increase.
β_4_	PA × Urban	Measures how the protection-abundance relationship varies as the proportion of nearby urban lands increases.
β_5_	PA × Agric	Measures how the protection-abundance relationship varies as the proportion of nearby agricultural lands increase.
β_6_	Savanna	Measures how average bird abundance varies as the proportion of savanna vegetation increases.

Biome is a major driver of bird diversity in the study area, which consisted of savanna and grassland, present in almost equal proportions (savanna occupies the northern 50% of the study area, and grassland the southern 50%). Including }{}{{\beta}_6} accounted for abundances of birds within the savanna biome. Only savanna was included in the model, as a covariate since the proportion of grassland is given by subtracting the proportion of savanna from 100%; including both would be redundant.

The Royle-Nichols model is related to the N-mixture model (Royle, 2004) that has recently been criticized for not being able to reliably separate abundance and detection (Barker et al., 2018, but see Kery, 2018). So, is the Royle-Nichols model appropriate for our data and would there be other approaches that would be more reliable in our situation? Our data clearly contain information on abundance and there are many studies before ours that have used the Southern African Bird Atlas data to analyse patterns in abundance (Huntley et al., 2012; e.g. Robertson et al., 1995). These studies generally used the ‘reporting rate,’ that is the proportion of checklists that report a given species, as a measure of abundance with the argument that the spatial variation in abundance is the main driver of detection probability. The Royle-Nichols model formalises this relationship and yields abundance estimates that are mathematically related to reporting rates (Altwegg & Nichols, 2019). However, the Royle-Nichols model allows us to account for other variables that affect detection probability. In our case, this was particularly the number of hours spent birding for each checklist, which we expected to affect the detection probability. The Royle-Nichols model should therefore be at least as reliable at recovering spatial patterns in abundance as an analysis of raw reporting rates. Barker et al. (2018) suggest using Poisson regression, which is not appropriate in our case as we do not have counts. We therefore decided to use the Royle-Nichols model and are confident that it reliably recovers patterns in abundance, which our study focuses on. We are less confident in the absolute abundance estimates and do not interpret these.

An important major assumption of the Royle-Nichols model is that the populations under study are closed (i.e. species abundance does not change markedly over the course of the study period). In reality, bird abundances do change over time, and thus, the closure assumption is usually violated to some degree. To minimize violation of this model assumption, we chose a relatively short time window of two years, over which these common, resident bird populations are relatively stable. Our main results should be relatively robust to violations of the closure assumption because they rely on comparing relative abundance estimates, and not absolute ones (Barker et al., 2018). We fitted abundance models to the data for each species separately, using package ‘unmarked’ (Fiske & Chandler, 2011) in program R (R Development Core Team, 2017).

#### Interpretations of model beta coefficients

In the absence of urban and agricultural area, the relationship between the proportion of protected area and bird abundance in a pentad is represented by }{}{{\beta}_1} (protection–abundance relationship). Our primary aim was to examine how this relationship changed with increasing proportions of urban and agricultural area in the same pentad. Therefore, the model parameters }{}{{\beta}_4} and }{}{{\beta}_5} (Eq. (5)), which estimate the effects of the interactions between the protected area and urban (}{}{{\beta}_4}) or agricultural (}{}{{\beta}_5}) area within the same pentad were of most interest. They indicate the degree to which the slope for the linear protection–abundance relationship changed when the proportion of urban (}{}{{\beta}_4}) or agricultural (}{}{{\beta}_5}) area within the same pentad changes. Species with a positive }{}{{\beta}_4} and }{}{{\beta}_5} value indicate that the slope of the protection–abundance relationship increases (i.e. becomes more positive) as the amount of urban or agricultural area increases within the pentad, meaning that the effect of protected area on the abundance of birds becomes more positive when urban or agricultural area neighbours protected area. The opposite is true for negative }{}{{\beta}_4} and }{}{{\beta}_5} values. We further examined variation in }{}{{\beta}_4} and }{}{{\beta}_5} through guilds using simple data aggregation, and a hierarchical Bayesian analysis (see sections below).

*For what percentage of species does the protection–abundance relationship increase or decrease with increasing proportions of urban and agricultural land in the same pentad?*

**Figure 2:**
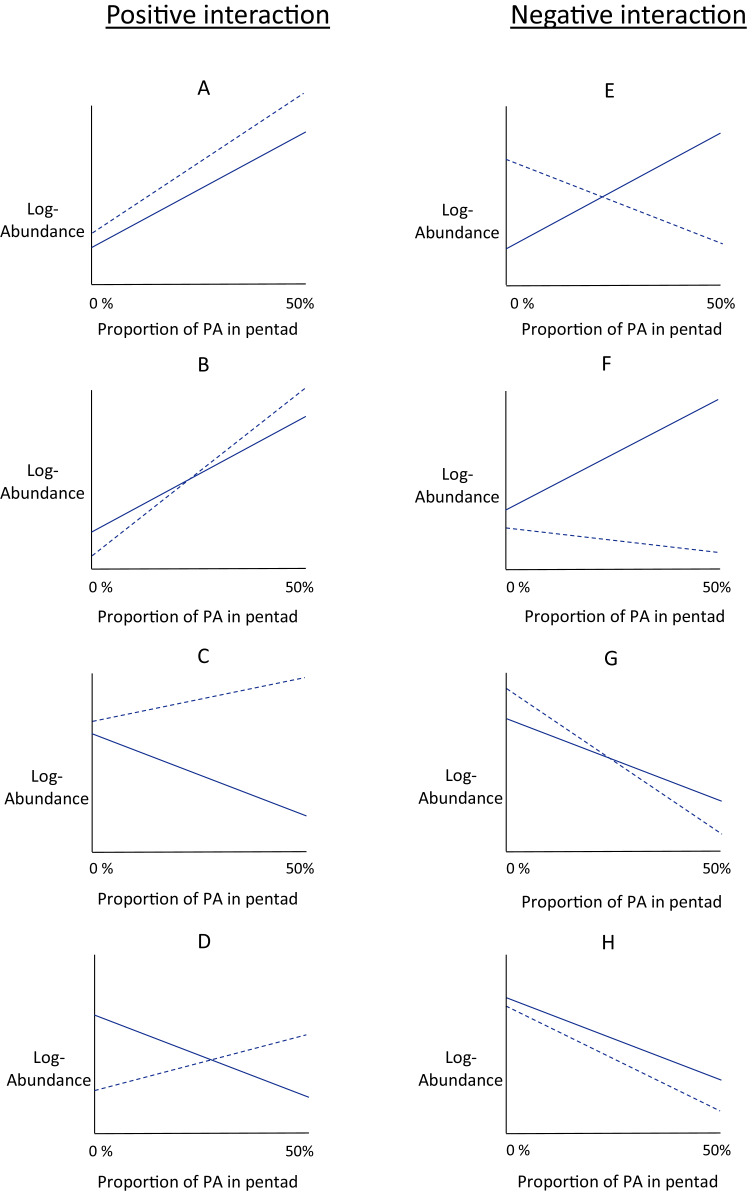
Conceptual interaction scenarios. Conceptual scenarios showing some possible interactions between protected area, log-abundance of birds, and one of the land-use types (agriculture or urban) within the same pentad. The conceptual graphs are identical for urban and agricultural land-use type. Log - abundance (*y*-axis) is modelled as a linear function of the proportion of protected area (‘PA’ on the *x*-axis) within a pentad. This is the protection-abundance relationship (the slope of this relationship is estimated by β_1_ in Eq. 5), and is indicated by the solid line. The dotted line indicates how this relationship is modified when either agricultural or urban area occupy 50% of the pentad. Positive interactions, that is the protection-abundance relationship becomes more positive as either agricultural or urban area increase, are in the left-hand column (scenarios A–D). Negative interactions, that is the protection–abundance relationship becomes more negative as either agricultural or urban area increase, are in the right-hand column (scenarios E–F).

In response to increasing proportions of urban or agricultural area nearby protected area, the slope of the protection–abundance relationship may increase (a significantly steeper slope describing the protection–abundance relationship), decrease (a significantly less steep slope), remain the same (a slope with no significant change), or may even change sign completely (change from a positive slope to a negative slope, or vice versa). The type of change in the protection–abundance relationship for each species is indicated by the estimates for the interaction coefficients (}{}{{\beta}_4} and }{}{{\beta}_5}), specified in Eq. (5). Interpreting the estimates for these coefficients provides a good understanding of how bird abundances are predicted to change within protected area with increasing proportions of agricultural and urban area near protected area. However, a more thorough understanding of how this occurs is gained from interpreting these interaction coefficients with the estimates for the other land-use type covariates that pertain to land-use types (}{}{{\beta}_0} -}{}{{\beta}_3}, main effects in Eq. (5)). Thus, we used the main and interaction effects that pertain to land-use types (}{}{{\beta}_0} -}{}{\beta}_5}), and examined eight scenarios of the way in which urban and agricultural area near protected area could potentially influence the relationship between bird abundance and the proportion of protected area (Figs. 2A–2H). There are, of course, many more hypothetical scenarios that could be considered, but the ones considered in this study are those that best align with the ecological hypotheses presented earlier. Distinguishing between these scenarios allows for a better understanding of the overall fitted relationships of the main and interaction effects in Eq. (5) as estimated by the model.

Figure 2 is divided into two columns which show positive interactions (left-hand column) and negative interactions (right-hand column). With positive interactions, the protection–abundance relationship becomes more positive as urban or agricultural area within the same pentad increase, as illustrated by the dotted line having a more positive slope than the solid one. With negative interactions, the protection–abundance relationship becomes more negative as urban or agricultural area within the same pentad increase, as illustrated by the dotted line having a more negative slope than the solid line.

In scenarios where pentads are without protected area, local abundance is a function of the main effects of urban or agricultural area, illustrated by examining values of the y-intercepts, and relative to natural land. The intercept for the solid line indicates the average abundance for a land-use type scenario of 0% protected area, and 100% natural land. The intercept for the dotted line indicates the average abundance for a land-use type scenario of 0% protected area, and 50% either urban or agricultural area, and 50% natural land. Scenarios A, C, E and G show situations where the species is more abundant in urban/agriculture area (dotted line y-intercept) than in natural land (solid line y-intercept—positive main effect); and in scenarios B, D, F and H, species are less abundant (negative main effect).

We fitted an abundance model to each species using Eqs. (1)–(5). Then, we categorised each species into scenarios A–H (Fig. 2), based on the way the species reacted to increases in urban area and agricultural area that surrounded protected areas. As a single guild was assigned to each species, we counted the frequency of species within each guild that fell into each interaction scenario (A–H). This indicated the manner in which the protection–abundance relationship for each guild is expected to change given increases in urban/agricultural land near protected area (assuming all other factors in that relationship remain constant). This analysis counts only the frequency with which species’ protection–abundance relationship is predicted to increase or decrease, rather than the magnitude of this change; this is considered in the next section below.

*What is the average change in magnitude of the protection–abundance relationship with increasing proportions of urban and agricultural land in the same pentad?*

We used a hierarchical Bayesian analysis to estimate the average change in the magnitude of the average protection–abundance relationship for each guild, along with associated credible intervals. This analysis used the species-specific mean and standard error estimates for the interaction terms \hat \beta_*4*_ (urban) and \hat \beta_*5*_ (agriculture) from Eq. (5) in the Royle-Nichols model of abundance to estimate a mean \hat \beta_*4*_ and \hat \beta_*5*_, and associated credible intervals, for each guild. The basic structure of this model was similar to a linear mixed-effects model, with guild as a random factor and normally distributed errors. However, instead of treating the mean interaction estimates as if they were observed values, we modelled them as coming from a normal distribution using the means and standard errors as estimated by the Royle-Nichols model of abundance. This approach is similar to the analysis described in McCarthy & Masters (2005) (see also Lloyd et al., 2014). We used non-informative priors for the mean interaction response per guild. We implemented this in the program to do still JAGS with 50,000 iterations and 25,000 burn in and 3 MCMC chains. The Gelman-Rubin diagnostic indicated that this model converged, and all *R*-hat values were <1.01. The JAGS code for this model is provided in the Appendix (Model A1).

## Results

### Royle-Nichols abundance models

*For what percentage of species does the protection–abundance relationship increase or decrease with increasing proportions of urban and agricultural land in the same pentad?*

The protection–abundance relationship became more positive, and hence the overall abundance per pentad was expected to increase for 58% of all species as urban area near protected areas increased in proportion (Table 2A, row 8). The protection–abundance relationship became more positive for 49% of all species as agricultural area near protected area increased in proportion (Table 2B, row 8).

**Table 2:** Percentage of species within each interaction scenario. Percentage of species of each guild that fall into the interaction scenarios (A–H, Fig. 2) based on the protected areas × urban land-use interaction (1), and the protected areas × agricultural land-use interaction (2). Scenarios A, B, C and D indicate situations where the protection-abundance relationship becomes more positive with increasing agricultural or urban area in the same pentad (termed ‘positive interaction scenarios’). Scenarios E, F, G and H indicate situations where the protection-abundance relationship becomes more negative within increasing surrounding agricultural or urban area (termed ‘negative interaction scenarios’). Each row sums to 100%. Column ‘Total’ under ‘Positive interaction scenarios’ heading sums the percentage of scenarios A–H for each feeding-guild, and likewise, column ‘Total’ under ‘Negative interaction scenarios’ heading sums the percentage of scenarios E–H.

Row	Feeding guild	Positive interaction scenarios					Negative interaction scenarios				
		A	B	C	D	Total	E	F	G	H	Total
Part A: Urban land-use type											
1	Frugivores	11	23	0	11	45	11	0	33	11	55
2	Gleaners	3	3	31	31	68	7	3	22	0	32
3	Granivores	0	4	13	43	60	6	10	15	9	40
4	Ground-feeders	6	6	24	24	60	24	3	8	5	40
5	Hawkers	0	18	9	27	54	18	10	18	0	46
6	Predators	11	0	16	32	59	15	15	11	0	41
7	Vegivores	11	5	11	16	43	37	0	20	0	57
8	Overall	5	6	18	29	58	17	6	15	4	42
Part B: Agriculture land-use type											
1	Frugivores	0	0	22	34	56	44	0	0	0	44
2	Gleaners	0	0	28	41	69	10	7	14	0	31
3	Granivores	0	4	2	19	25	9	9	44	13	75
4	Ground-feeders	5	5	15	28	53	24	5	10	8	47
5	Hawkers	0	0	0	36	36	36	9	10	9	64
6	Predators	5	0	11	36	52	26	11	11	0	48
7	Vegivores	11	11	5	25	52	32	0	11	5	48
8	Overall	3	4	12	30	49	21	6	18	6	51

Of the positive interaction scenarios (A, B, C, D; Fig. 2) C and D were observed most frequently in total, for increases in proportion of both urban (Table 2A, row 8) and agricultural area (Table 2B, row 8). In total, scenarios C and D together comprise 81% of all the positive interaction scenarios as urban area increased, 86% of all the positive interaction scenarios as agricultural area increased. Scenario D, in particular, contributed 63% and 86% of the positive interaction scenarios of species for which the protected area by urban land use interaction or the protected area by agricultural land use interaction was significantly different to zero.

Of the negative interaction scenarios (E, F, G, H; Fig. 2) E and G were observed most frequently in total, for increases in proportion of both the urban (Table 2A, row 8) and agricultural area (Table 2B, row 8). In total, scenarios E and G made up 75% of all negative interaction scenarios as both urban and agricultural area increased. In particular, scenario E contributed 64% and 69% respectively to all the negative interaction scenarios of species for which the protected area by urban land use interaction or protected area by agricultural land use interaction was significantly different to zero (Table A3, panel 2, Appendix).

*What is the average change in magnitude of the protection–abundance relationship with increasing proportions of urban and agricultural land in the same pentad?*

**Figure 3:**
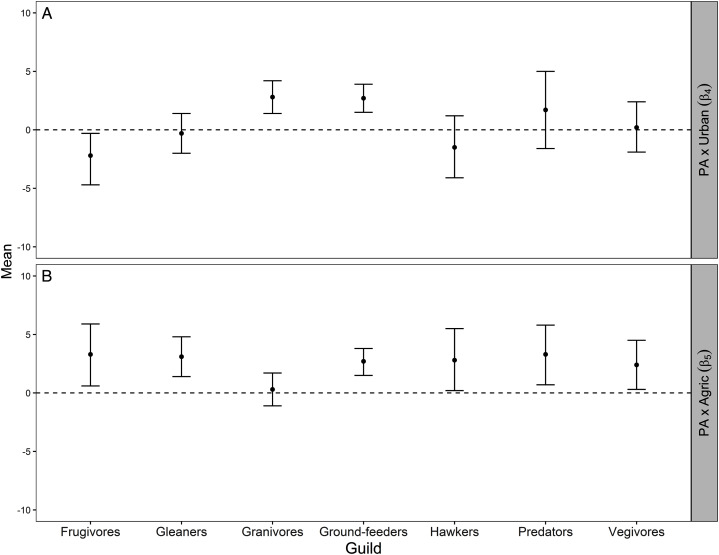
Mean and 95% credible interval for Bayesian meta-analysis. Mean and 95% credible interval indicating how the average protection-abundance relationship for each guild is modified by an increasing proportion of urban (β_4_; A) and agricultural (β_5_; B) area, as estimated by the Bayesian hierarchical analysis. These are the interaction effects β_4_ and β_5_ specified in Eq. 5. Positive values indicated the protection–abundance relationship becomes more positive for each guild, on average, as the proportion of urban or agricultural area in the pentad increases. Negative values indicated the opposite. β_4_ and β_5_ values estimated by the Royle-Nichols model of abundance fitted once for each of the 198 species. Confidence intervals that do not overlap zero indicate a statistically significant effect for either β_4_ and β_5_ whilst confidence intervals that overlap zero indicate no statistical significance.

For the granivores and ground-feeder guilds, the average protection–abundance relationship became significantly more positive with a higher proportion of urban area in the same pentad, whilst this relationship became more negative for frugivores, and neither more positive nor more negative for the remaining four guilds (gleaners, hawkers, predators, and vegivores), Fig. 3A.

As the proportion of agricultural area increased within pentads, the average protection–abundance relationship became significantly more positive for six of the seven guilds: frugivores, vegivores, predators, gleaners, ground feeders and hawkers. For granivores, the average change in the protection–abundance relationship was close to zero, and non-significant (Fig. 3B).

Figure A3 (Appendix) presents a visual summary of the parameter estimates across guilds and Table A2 (Appendix) shows model results for each species within each guild.

## Discussion

Protected areas are a key tool for biodiversity conservation. However, there are concerns that the ecological effectiveness of protected areas (defined in this study as the degree to which protected areas conserve species and habitats) is influenced by nearby land-use types in the landscape (Cottee-Jones et al., 2015; DeFries, Karanth & Pareeth, 2010; Santos et al., 2008; Thomas et al., 2012). In this study, we examined to what degree this concern applies to a large group of common species across many protected areas over a heterogeneous landscape in the greater Gauteng region of South Africa. Using abundance models, which are an extension of occupancy models (Royle & Nichols, 2003), we modelled how urban and agricultural land-use types near to protected areas affected the relationship between protected areas and bird abundances (protection–abundance relationship). Our main results suggest that urban and agricultural area near protected areas affect the protection–abundance relationship, but that the magnitude and direction of this effect differs between land-use types and guilds. Our results further suggest that protected areas do not function in isolation, but rather, they must be considered as a constituent component of the greater landscape.

### The effect of agricultural areas near protected areas on the protection–abundance relationship

The average protection–abundance relationship became more positive in response to an increasing proportion of agricultural area within the same pentad for six of the seven guilds (frugivores, vegivores, predators, gleaners, ground-feeders and hawkers), and none decreased significantly (Fig. 3). We initially hypothesised that the protection–abundance relationship for ground-feeders, hawkers and predators would increase (become more positive) as agricultural and urban areas near protected areas increase in proportion; our results thus concur with this hypothesis. For these guilds, interaction cases C and D (Fig. 2) were the most common ways in which the average protection–abundance relationship increased (Table 2). C and D represent cases in which a negative protection–abundance relationship becomes positive as agricultural area near protected area increases in proportion. These results suggest that in the absence of agricultural land use (and for a scenario where protected areas are neighboured by only natural land-use type), guilds ground-feeders, hawkers, and predators are more abundant outside protected areas, and thus, more abundant within the natural land, compared to inside protected areas. However, as the proportion of agricultural land use surrounding protected areas increases, guilds ground-feeders, hawkers, and predators become more abundant inside protected areas, compared to outside. This result shows a synergistic relationship between protected areas and agricultural land, and on average, these guilds prefer protected areas to agricultural land. This result could possibly be due to the activities associated with farming practices which decrease the quality of the soil, air, and water within entire landscapes, rendering agricultural area less suitable for a broad range of bird guilds (Billeter et al., 2008; Stoate et al., 2001).

### The effect of urban areas on the protection–abundance relationship

We predicted the average protection–abundance relationship would increase for the insect eating guilds (ground-feeders, hawkers and gleaners) as the proportion of urban areas near protected areas increased, and decrease for granivores. However, our results only partially supported our hypothesis; on average, guilds ground-feeder and granivores became significantly more positive in their average protection–abundance relationship, whilst this relationship decreased significantly for frugivorous species (Fig. 3A).

The most frequent scenario by which the average protection–abundance relationship increased for the ground-feeder guild was via interaction cases C and D, and via cases D for the granivorous guild. C and D describe a scenario where a negative protection–abundance relationship becomes more positive as urban area increases near protected area. These results suggest that in the absence of urban land near protected areas, ground-feeder and granivorous guilds are more abundant inside protected areas, compared to outside. However, as the proportion of urban area near protected areas increases, ground-feeder and granivorous guilds become more abundant inside protected areas. These results indicate a synergistic relationship between protected areas and urban land for the ground-feeder and granivorous guilds. Our results could possibly be due to the negative effects of dense urban areas on wildlife, such as persecution, predation, or pollution (Blair, 1996), or even the design and use of the urban areas by people (Paker et al., 2014).

## Conclusion

In conclusion, we found strong evidence that the ecological effectiveness of protected areas (protection–abundance relationship) was affected by the proportion of urban and agricultural areas. Agricultural areas near protected areas increased the average ecological effectiveness to a higher degree than did urban areas near protected area. On average the protection–abundance relationship of six guilds (frugivores, gleaners, ground-feeders, hawkers, predators and vegivores) increased as the proportion of agriculture and protected area increased. Conversely, as urban area near protected area increased in proportion, only two guilds increased in their average protection–abundance relationship (granivores and ground-feeders), whilst this relationship was unchanged for the remaining four guilds (gleaners, hawkers, predators and vegivores), and decreased for frugivores. The major way in which near urban and agricultural land changed the bird abundance inside protected area was by a negative protection–abundance relationship becoming less negative. A future research direction, therefore, is to reveal the exact mechanisms that underpin this transition. Our results indicate that a heterogeneous landscape which includes protected, urban, and agricultural areas, rather than uniform habitats of single use, may benefit biodiversity, and in doing so may increase the ecological effectiveness of protected areas.

## Supplemental Information

10.7717/peerj.10719/supp-1 Supplemental Information 1 Appendix. Additional study area maps, code to perform the bayesian meta-analysis, and the model estimates for the Royle-Nichols model of abundance.

10.7717/peerj.10719/supp-2 Supplemental Information 2 Appendix (for Bayesian meta-analysis). Needed for running the R code and conducting the Bayesian meta-analysis that produces Fig. 3.

10.7717/peerj.10719/supp-3 Supplemental Information 3 R code to perform Bayesian meta-analysis that produces Figure 3. To run the code, the package ‘R2jags’ must be installed already.

10.7717/peerj.10719/supp-4 Supplemental Information 4 Detection/non-detection data for the 198 species. Data of detection or non-detection of species over the study area. Individual species are the columns, and each grid cell is a row. Where a species is detected in a grid cell, its species number is recorded. These species numbers map back to the table of species’ numbers, common names and Latin names, included as a separate dataset.

10.7717/peerj.10719/supp-5 Supplemental Information 5 Species’ Names. Table of species’ common names, Latin names, and identification numbers. Only the species’ number is included in the main dataset (‘spp_detection_data.txt’), and this table maps the number to the species’ name.

## Additional Information and Declarations

### Data Availability

The following information was supplied regarding data availability: Raw data and analysis code are available as Supplemental Files.
