# Supplementary material for: Why a landscape view is important: nearby urban and agricultural land affects bird abundances in protected areas
Source: PeerJ. 2021 Jul 28;9:e10719. doi: 10.7717/peerj.10719 (PMC8325429; doi:10.7717/peerj.10719)
Supplement: Supplemental Information 1 — Additional study area maps, code to perform the bayesian meta-analysis, and the model estimates for the Royle-Nichols model of abundance. [file peerj-09-10719-s001.docx]

**APPENDIX:** Supplementary material


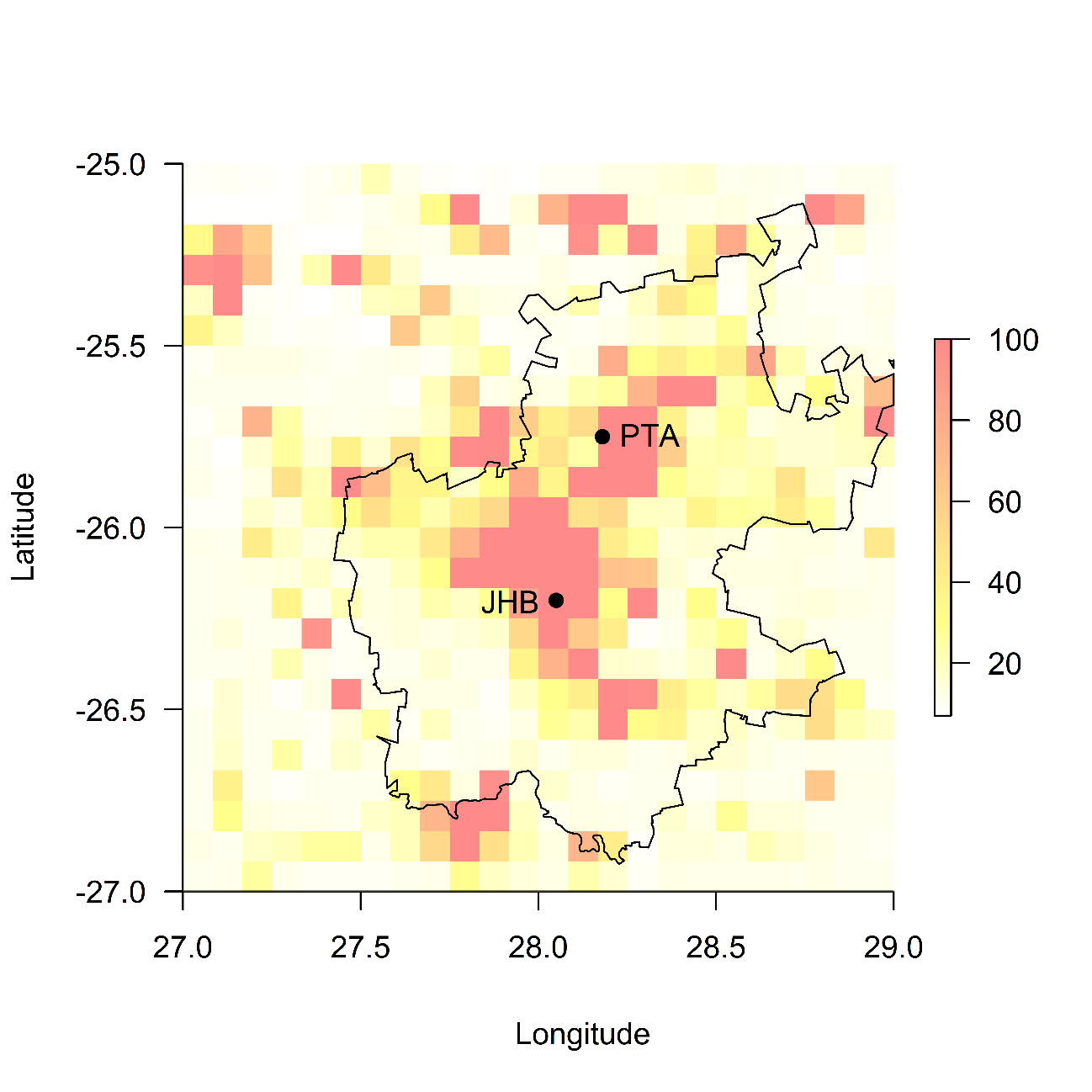


**Figure A1.** Checklists submitted per pentad across the study region. The study area comprised the greater Gauteng region, which included the cities of Johannesburg and Pretoria (respectively shown as ‘JHB’ and ‘PTA’). Each pentad is 5’ x 5’ in dimension (unit is arcminutes; approximately 61 km^2^ in area), and the number of checklists submitted per pentad corresponds to the colour of the pentad; a darker colour indicates a higher number of checklist submissions. For the analysis, we capped the maximum number of checklist submissions at 100.

**
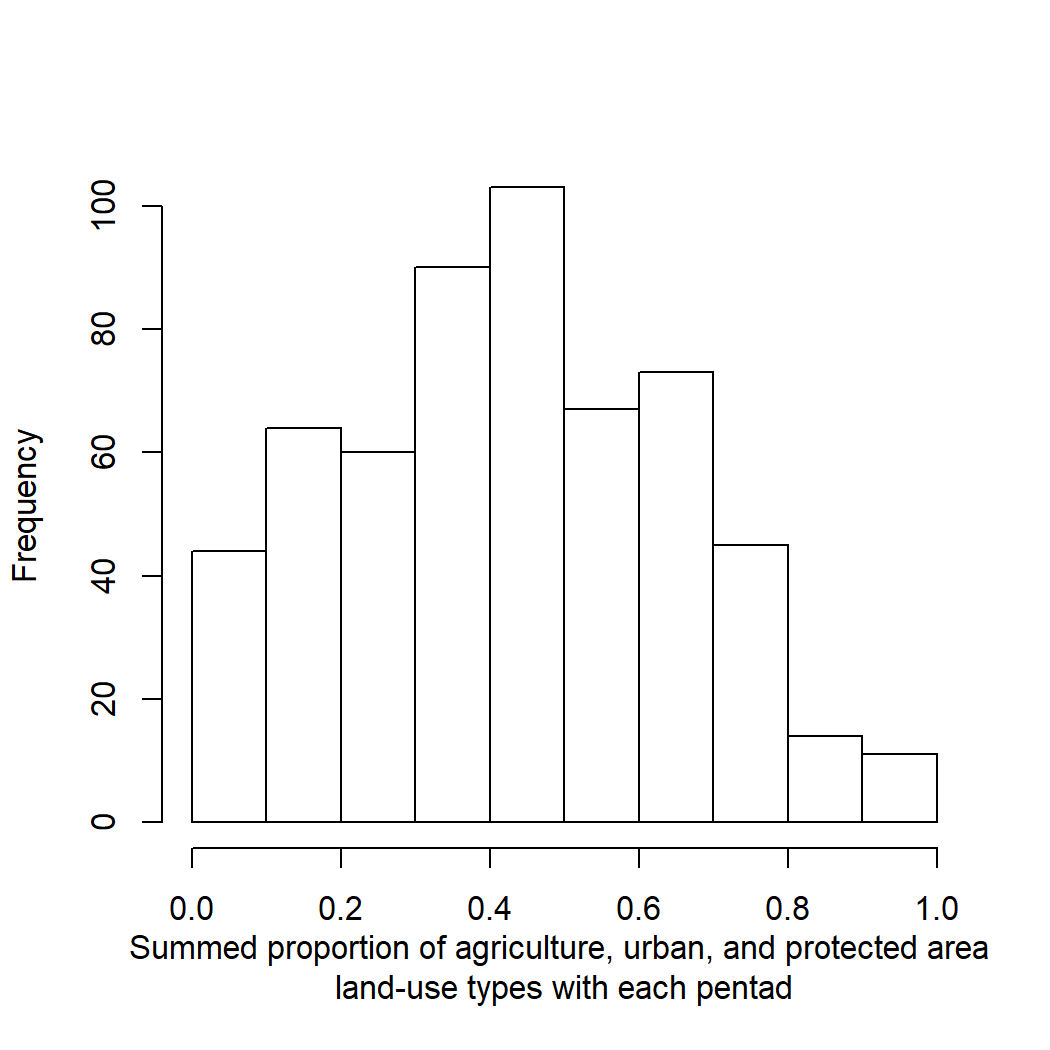
**

**Figure A2.** Histogram showing summed proportion of land-use types agriculture, urban, and protected area for each pentad over the study area.

**Table A1.** Variance Inflation Factors (VIF) for each land-use type used in the Royle-Nichols abundance model. VIFs calculated from the land-use proportion within each of the 576 pentads (5’ × 5’ grid cell, approximately 61 km^2^) which makes up the study area. Generally, a VIF statistic of below five indicates no multicollinearity.

| Land-use type | VIF |
| --- | --- |
| Agriculture | 1.60 |
| Urban | 1.14 |
| Protected areas | 1.13 |

**Model A1.** JAGS model code for Bayesian analysis, describing the average β_4_ and β_5_ for each guild, along with associated error.

The model below codes the Bayesian analysis, the results of which produced figure 3. This model estimates the average $\hat{\beta}$_4_ and $\hat{\beta}$_5_ for each guild, along with standard error. $\hat{\beta}$_4_ and $\hat{\beta}$_5_ are the coefficients of the interaction terms in equation 5 in the methods text: $\hat{\beta}$_4_ represents the protected areas $\times$ urban interaction, and $\hat{\beta}$_5_ represents the protected areas $\times$ agriculture interaction. The model was fitted once for each beta (the model is identical for either beta).

# Library

library(R2jags)

# Read in data

app <- read.csv(paste0(getwd(), "/appendix.csv"), header = T)

# Write the model in BUGS Language as a function

lm1_jags = function() {

# Likelihood

for(i in 1:n) {

betas[i] ~ dnorm(mu[i], tau.obs[i])

tau.obs[i] <- pow(se[i], -2)

mu[i] ~ dnorm(mu.int[fg[i]], tau.species)

}

# Priors

# - Beta estimates across each FG

for(f in 1:7) {

mu.int[f] ~ dnorm(0, 0.0001)

}

# - Error associated with beta estimates across each FG

sd.species ~ dunif(0, 100)

tau.species <- pow(sd.species, -2)

}

# Specify initial values

init_values <- function() {

list(mu.int = rnorm(7,1))

}

# Names of parameters to save

params = "mu.int"

# Bundle the data

# Agricultural data (beta 4)

test_data_agric <- list(

'betas' = app$Paagric,

'se' = app$Paagric_SE,

'fg' = as.numeric(app$FG),

'n' = dim(app)[1]

)

# Urban data

test_data_urban <- list(

'betas' = app$Paurban,

'se' = app$Paurban_SE,

'fg' = as.numeric(app$FG),

'n' = dim(app)[1]

)

# Fit the models

# Agricultural land use meta-analysis

fit_lm1_agric <- jags(data = test_data_agric,

parameters.to.save = params,

model.file = lm1_jags,

n.chains = 3,

n.iter = 50000,

n.burnin = 25000,

n.thin = 10,

DIC = F

)

# Urban land use meta-analysis

fit_lm1_urban <- jags(data = test_data_urban,

parameters.to.save = params,

model.file = lm1_jags,

n.chains = 3,

n.iter = 50000,

n.burnin = 25000,

n.thin = 10,

DIC = F

)


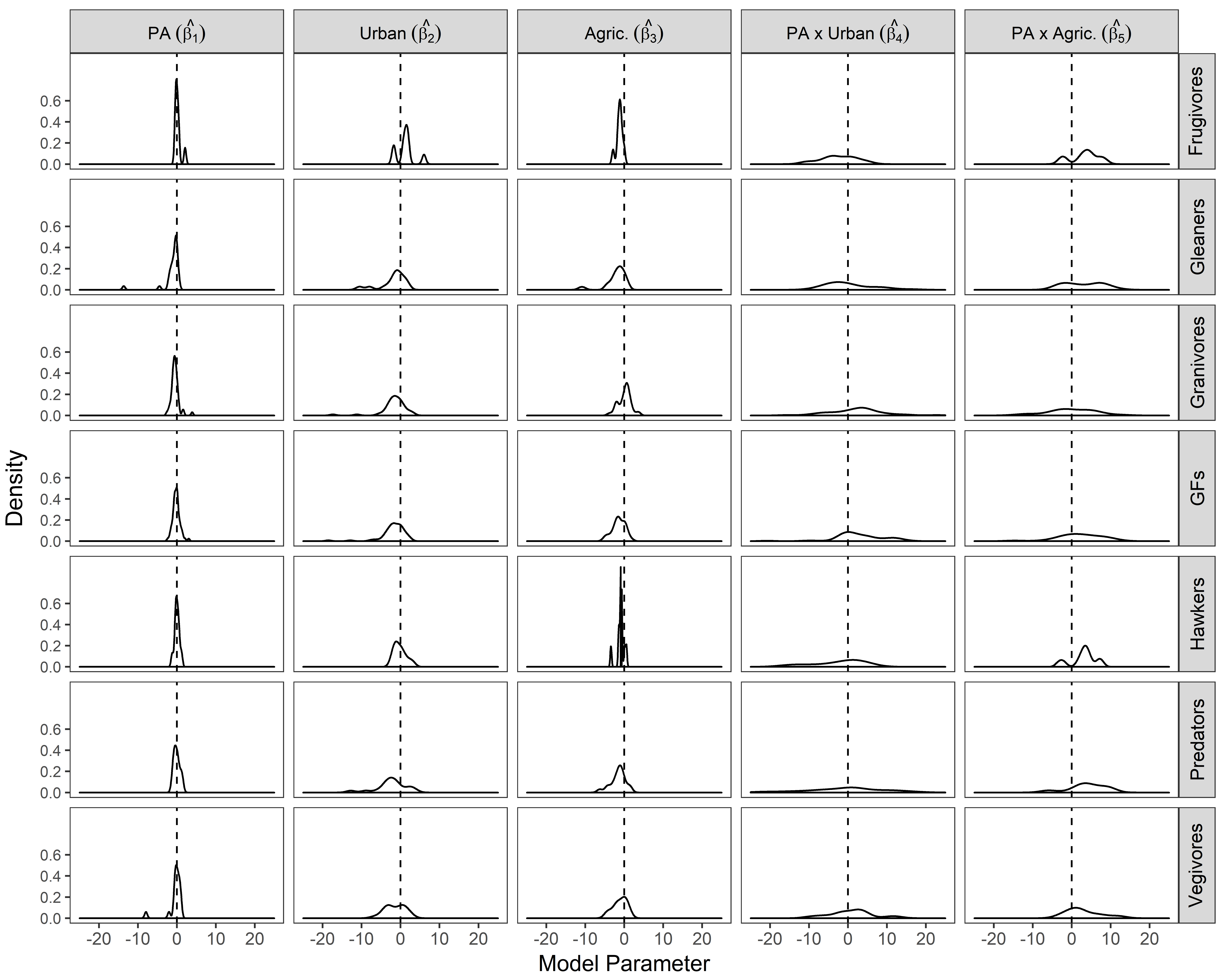


**Figure A3.** Density plots of model coefficients for 196 bird species as estimated by the Royle-Nichols model of abundance (equation 5, Methods; abundance component). Model parameters are labelled by columns, and the guild to which each species belongs is labelled by rows. Only mean model estimates are shown (standard deviations for each mean estimate are omitted). The model estimates abundance of birds per pentad as a function of the proportion of a pentad occupied by protected areas (‘PA’), agricultural land (‘Agric.’), urban land ‘(Urban’), and their interactions. A single model was fitted for each of the 196 species independently, and the study area was the greater Gauteng region in South Africa. ‘GF’ refers to the ground-feeding guild (see methods text for description of guilds).

**Table A2.** Mean and standard errors of model parameters estimated by the Royle-Nichols model of abundance, fitted independently for each of the 196 common, resident species. The estimated $\hat{\beta}$ here refer to those specified in equation 5 of the main methods text, and estimates are on the log scale (estimates for the detection components have been omitted). ‘Agricultural interaction-scenario’, and ‘Urban interaction-scenario’ refer to which of the interaction scenarios, as defined in Figure 2 of the main text, a species falls. ‘Guild’ refers to the guild which has been assigned to each species (‘GF’ refers to the guild ground-feeder). When fitting the models, all covariates were standardized to a mean of zero and a standard deviation of one, to ensure model convergence. ‘PA’ represents Protected Areas land-use type, and ‘Agric. and ‘Urb.’ represent Agricultural and Urban land-use types respectively.

| Guild | Species | Abundance model estimates (log scale) | | | | | | | Agric. interaction-scenario | Urban interaction-scenario |
| --- | --- | --- | --- | --- | --- | --- | --- | --- | --- | --- |
|  |  | Intercept  ($\hat{\beta}$*_0_*) | PA  ($\hat{\beta}$*_1_*) | Urban  ($\hat{\beta}$*_2_*) | Agric.  ($\hat{\beta}$*_3_*) | PA × Urban  ($\hat{\beta}$*_4_*) | PA × Agric.  ($\hat{\beta}$*_5_*) | Savanna  ($\hat{\beta}$*_6_*) |  |  |
|  |  |  |  |  |  |  |  |  |  |  |
| Frugivore | Acacia Pied Barbet *(Tricholaema leucomelas)* | 0.61 ± 0.20 | -0.42 ± 0.42 | -1.68 ± 0.48 | -0.87 ± 0.38 | 4.81 ± 3.42 | -2.21 ± 2.56 | 0.32 ± 0.15 | C | D |
| Frugivore | African Olive-Pigeon (*Columba arquatrix*) | -1.21 ± 0.30 | 2.11 ± 0.67 | 6.01 ± 0.37 | -0.03 ± 0.67 | -10.54 ± 5.20 | 4.99 ± 3.57 | -1.23 ± 0.24 | E | B |
| Frugivore | Black-collared Barbet (*Lybius torquatus*) | 1.22 ± 0.13 | -0.03 ± 0.26 | 0.82 ± 0.23 | -1.59 ± 0.27 | 0.27 ± 2.09 | 3.02 ± 1.46 | 0.29 ± 0.10 | D | H |
| Frugivore | Cape Glossy Starling (*Lamprotornis nitens*) | 1.51 ± 0.12 | -0.19 ± 0.24 | 0.72 ± 0.20 | -1.10 ± 0.22 | -4.13 ± 2.19 | -2.32 ± 1.54 | 0.30 ± 0.08 | C | G |
| Frugivore | Crested Barbet (*Trachyphonus vaillantii*) | 1.27 ± 0.12 | -0.27 ± 0.25 | 1.36 ± 0.20 | -1.05 ± 0.23 | -2.91 ± 2.19 | 4.43 ± 1.38 | 0.24 ± 0.09 | D | G |
| Frugivore | Dark-capped Bulbul (*Pycnonotus tricolor*) | 1.21 ± 0.11 | 0.39 ± 0.21 | 1.84 ± 0.18 | -1.25 ± 0.22 | -4.22 ± 1.90 | 3.99 ± 1.15 | 0.54 ± 0.08 | E | B |
| Frugivore | Red-faced Mousebird (*Urocolius indicus*) | 1.01 ± 0.14 | -0.27 ± 0.29 | 1.57 ± 0.21 | -0.54 ± 0.25 | -6.64 ± 2.66 | 2.16 ± 1.55 | 0.38 ± 0.09 | D | G |
| Frugivore | Red-winged Starling (*Onychognathus morio*) | 0.11 ± 0.22 | 0.44 ± 0.33 | 1.90 ± 0.33 | -2.88 ± 0.53 | 0.19 ± 2.51 | 8.33 ± 1.84 | 0.57 ± 0.17 | E | A |
| Frugivore | Yellow-fronted Tinkerbird (*Pogoniulus chrysoconus*) | -1.87 ± 0.33 | 0.19 ± 0.32 | -1.83 ± 0.73 | -1.66 ± 0.56 | 2.02 ± 3.63 | 7.20 ± 1.75 | 2.90 ± 0.30 | E | E |
| Gleaner | Ashy Tit (*Parus cinerascens*) | -1.80 ± 0.50 | -1.60 ± 1.13 | -0.74 ± 1.05 | -1.46 ± 1.02 | -2.07 ± 9.70 | 2.43 ± 6.16 | 1.56 ± 0.41 | C | C |
| Gleaner | Barred Wren-Warbler (*Calamonastes fasciolatus*) | -7.54 ± 2.14 | -1.23 ± 1.07 | -2.90 ± 1.75 | 0.76 ± 0.92 | 11.10 ± 7.76 | -2.78 ± 5.78 | 7.08 ± 2.13 | G | D |
| Gleaner | Bar-throated Apalis (*Apalis thoracica*) | 0.07 ± 0.23 | 0.55 ± 0.36 | 0.86 ± 0.41 | -1.80 ± 0.52 | 0.87 ± 3.15 | 7.72 ± 1.86 | 0.28 ± 0.18 | E | A |
| Gleaner | Black-backed Puffback (*Dryoscopus cubla*) | -0.51 ± 0.23 | -0.42 ± 0.31 | 0.46 ± 0.39 | -2.71 ± 0.50 | -5.33 ± 3.16 | 8.17 ± 1.69 | 2.08 ± 0.20 | D | G |
| Gleaner | Black-chested Prinia (*Prinia flavicans*) | 1.69 ± 0.12 | -1.14 ± 0.38 | -1.10 ± 0.26 | 0.16 ± 0.22 | 3.58 ± 2.85 | -2.49 ± 2.04 | -0.24 ± 0.09 | G | D |
| Gleaner | Black-headed Oriole (*Oriolus larvatus*) | 0.02 ± 0.23 | -0.16 ± 0.30 | -1.37 ± 0.51 | -2.54 ± 0.50 | -2.20 ± 3.52 | 7.16 ± 1.70 | 1.65 ± 0.19 | D | C |
| Gleaner | Brubru Brubru (*Nilaus afer*) | -0.67 ± 0.31 | -1.24 ± 0.40 | -4.54 ± 0.89 | -2.48 ± 0.55 | 7.58 ± 3.95 | -2.07 ± 2.92 | 2.63 ± 0.27 | C | D |
| Gleaner | Cape Penduline-Tit (*Anthoscopus minutus*) | -5.14 ± 1.31 | -1.48 ± 1.29 | -1.59 ± 1.62 | 0.59 ± 1.24 | 7.32 ± 9.11 | -1.39 ± 6.93 | 4.91 ± 1.27 | G | D |
| Gleaner | Cardinal Woodpecker (*Dendropicos fuscescens*) | 0.76 ± 0.21 | -0.57 ± 0.33 | 0.01 ± 0.38 | -3.10 ± 0.49 | -3.97 ± 3.31 | 6.03 ± 1.98 | 0.89 ± 0.16 | D | G |
| Gleaner | Chestnut-vented Tit-Babbler (*Parisoma subcaeruleum*) | 0.29 ± 0.17 | -0.20 ± 0.27 | -1.12 ± 0.37 | -0.89 ± 0.32 | -2.44 ± 3.03 | -3.81 ± 1.88 | 1.47 ± 0.13 | C | C |
| Gleaner | Chinspot Batis (*Batis molitor*) | -0.41 ± 0.21 | -0.25 ± 0.23 | -1.63 ± 0.42 | -1.77 ± 0.37 | 0.69 ± 2.43 | 1.22 ± 1.51 | 2.67 ± 0.18 | D | D |
| Gleaner | Common Scimitarbill (*Rhinopomastus cyanmelas*) | -1.11 ± 0.48 | 0.38 ± 0.74 | -0.35 ± 0.92 | -0.68 ± 1.05 | 1.38 ± 6.54 | -2.21 ± 5.09 | 0.56 ± 0.38 | F | E |
| Gleaner | Crimson-breasted Shrike (*Laniarius atrococcineus*) | -1.27 ± 0.24 | 0.02 ± 0.28 | -1.89 ± 0.54 | -0.19 ± 0.38 | -2.43 ± 3.44 | -3.11 ± 1.91 | 2.85 ± 0.21 | F | F |
| Gleaner | Desert Cisticola (*Cisticola aridulus*) | 0.66 ± 0.22 | -0.18 ± 0.43 | -2.69 ± 0.59 | -0.23 ± 0.43 | -0.33 ± 5.10 | -1.87 ± 2.69 | 0.20 ± 0.17 | C | C |
| Gleaner | Golden-tailed Woodpecker (*Campethera abingoni*) | -0.15 ± 0.26 | -1.06 ± 0.40 | -0.64 ± 0.50 | -2.71 ± 0.58 | 1.13 ± 3.40 | 10.11 ± 2.07 | 1.81 ± 0.22 | D | C |
| Gleaner | Green Wood-Hoopoe (*Phoeniculus purpureus*) | 1.04 ± 0.16 | -0.82 ± 0.36 | 1.52 ± 0.24 | -1.25 ± 0.30 | -4.67 ± 2.97 | 6.78 ± 1.80 | 0.15 ± 0.11 | D | G |
| Gleaner | Grey Penduline-Tit (*Anthoscopus caroli*) | -27.80 ± 76.32 | -13.64 ± 8.53 | -124.46 ± 72.42 | -10.40 ± 5.29 | -6.87 ± 107.50 | 46.14 ± 36.32 | 30.30 ± 76.32 | D | C |
| Gleaner | Grey-headed Bush-Shrike (*Malaconotus blanchoti*) | -0.86 ± 0.33 | -0.26 ± 0.41 | -0.08 ± 0.60 | -2.52 ± 0.71 | -3.29 ± 4.39 | 7.47 ± 2.35 | 1.91 ± 0.27 | D | C |
| Gleaner | Klaas's Cuckoo (*Chrysococcyx klaas*) | -1.56 ± 0.41 | -0.17 ± 0.51 | 1.93 ± 0.48 | -1.04 ± 0.85 | -4.83 ± 4.27 | 2.16 ± 3.30 | 2.63 ± 0.33 | D | G |
| Gleaner | Lesser Honeyguide (*Indicator minor*) | 0.36 ± 0.24 | -0.74 ± 0.45 | 0.79 ± 0.40 | -1.72 ± 0.52 | -9.29 ± 4.81 | 4.73 ± 2.53 | 0.84 ± 0.18 | D | G |
| Gleaner | Long-billed Crombec (*Sylvietta rufescens*) | -1.69 ± 0.26 | -0.52 ± 0.24 | -1.01 ± 0.39 | -1.08 ± 0.35 | -5.22 ± 2.79 | -0.58 ± 1.60 | 4.13 ± 0.25 | C | C |
| Gleaner | Rattling Cisticola (*Cisticola chiniana*) | -1.73 ± 0.24 | -0.11 ± 0.26 | -0.57 ± 0.40 | -0.08 ± 0.35 | -3.14 ± 2.83 | -4.35 ± 1.84 | 3.62 ± 0.22 | C | C |
| Gleaner | Red-headed Weaver (*Anaplectes melanotis*) | -8.22 ± 4.82 | -2.03 ± 0.82 | -7.85 ± 3.32 | -11.32 ± 2.39 | -6.60 ± 16.69 | 3.62 ± 10.20 | 9.67 ± 4.84 | C | C |
| Gleaner | Southern Black Tit (*Parus niger*) | -3.41 ± 0.92 | -1.82 ± 0.56 | -10.55 ± 2.35 | -4.38 ± 0.89 | 9.62 ± 8.46 | 6.43 ± 3.44 | 4.96 ± 0.91 | D | D |
| Gleaner | Southern Boubou (*Laniarius ferrugineus*) | -0.41 ± 0.18 | 0.10 ± 0.28 | 1.52 ± 0.29 | -1.22 ± 0.36 | -0.57 ± 2.36 | 8.94 ± 1.33 | 1.45 ± 0.14 | E | B |
| Gleaner | Tawny-flanked Prinia (*Prinia subflava*) | 0.77 ± 0.14 | -0.19 ± 0.26 | 1.35 ± 0.23 | -1.27 ± 0.27 | -0.86 ± 2.16 | 6.82 ± 1.35 | 0.60 ± 0.10 | D | G |
| Gleaner | White-crested Helmet-Shrike (*Prionops plumatus*) | -4.46 ± 1.68 | -2.07 ± 1.07 | -8.02 ± 3.84 | -4.36 ± 1.66 | 8.22 ± 13.69 | 12.20 ± 5.65 | 4.94 ± 1.65 | D | D |
| Gleaner | Wing-snapping Cisticola (*Cisticola ayresii*) | 0.62 ± 0.27 | 0.12 ± 0.65 | -3.84 ± 0.83 | -0.22 ± 0.51 | 16.44 ± 4.75 | 1.35 ± 3.45 | -1.28 ± 0.24 | E | E |
| Gleaner | Yellow-bellied Eremomela (*Eremomela icteropygialis*) | -3.52 ± 2.93 | -4.47 ± 2.75 | -10.56 ± 6.55 | -4.29 ± 2.96 | 36.44 ± 18.47 | 8.90 ± 15.21 | 4.58 ± 2.75 | C | D |
| Gleaner | Zitting Cisticola (*Cisticola juncidis*) | 1.81 ± 0.15 | -0.47 ± 0.29 | -1.84 ± 0.28 | 0.60 ± 0.22 | 4.37 ± 2.56 | 0.20 ± 1.52 | -0.24 ± 0.09 | G | D |
| Granivore | African Firefinch (*Lagonosticta rubricata*) | -0.95 ± 0.49 | 0.10 ± 0.59 | -0.59 ± 0.90 | -1.91 ± 1.09 | -5.74 ± 6.79 | 4.92 ± 3.62 | 1.37 ± 0.39 | E | F |
| Granivore | African Quailfinch (*Ortygospiza atricollis*) | 1.43 ± 0.15 | 0.00 ± 0.35 | -4.97 ± 0.57 | 0.79 ± 0.27 | 6.43 ± 4.11 | -3.24 ± 2.08 | -0.64 ± 0.12 | B | E |
| Granivore | Black-faced Waxbill (*Estrilda erythronotos*) | -3.24 ± 0.53 | -0.08 ± 0.68 | -2.51 ± 1.31 | 1.19 ± 0.73 | 3.98 ± 6.52 | -11.96 ± 5.97 | 3.21 ± 0.48 | G | D |
| Granivore | Black-throated Canary (*Crithagra atrogularis*) | 1.49 ± 0.12 | -0.86 ± 0.35 | -0.93 ± 0.25 | 0.91 ± 0.20 | 4.50 ± 2.58 | -0.35 ± 1.68 | -0.20 ± 0.09 | G | D |
| Granivore | Blue Waxbill (*Uraeginthus angolensis*) | -0.55 ± 0.17 | -0.69 ± 0.23 | -1.16 ± 0.33 | -0.40 ± 0.27 | 3.71 ± 1.99 | 2.16 ± 1.29 | 3.00 ± 0.14 | D | D |
| Granivore | Bronze Mannikin (*Spermestes cucullatus*) | -1.19 ± 0.25 | -0.68 ± 0.56 | 3.33 ± 0.33 | -0.58 ± 0.56 | 1.81 ± 3.19 | 6.62 ± 2.71 | 1.32 ± 0.19 | D | H |
| Granivore | Cape Canary (*Serinus canicollis*) | -1.82 ± 0.81 | 1.49 ± 1.32 | 0.20 ± 1.62 | -2.22 ± 1.96 | -7.34 ± 16.54 | 8.49 ± 6.65 | -1.08 ± 0.72 | E | B |
| Granivore | Cape Sparrow (*Passer melanurus*) | 1.79 ± 0.10 | -1.63 ± 0.40 | 1.44 ± 0.16 | 1.02 ± 0.17 | 4.29 ± 2.32 | 2.77 ± 1.74 | -0.77 ± 0.08 | G | H |
| Granivore | Cape Turtle-Dove (*Streptopelia capicola*) | 1.89 ± 0.11 | -0.31 ± 0.26 | 0.04 ± 0.19 | 0.19 ± 0.19 | 0.36 ± 2.24 | -3.31 ± 1.52 | -0.36 ± 0.07 | G | G |
| Granivore | Chestnut-backed Sparrowlark (*Eremopterix leucot*is) | -2.57 ± 0.49 | -0.53 ± 1.58 | -3.05 ± 1.73 | 3.84 ± 0.83 | 6.63 ± 13.64 | -4.72 ± 6.83 | 0.77 ± 0.37 | G | D |
| Granivore | Cinnamon-breasted Bunting (*Emberiza tahapisi*) | 1.17 ± 0.20 | 0.03 ± 0.28 | -2.62 ± 0.51 | -2.20 ± 0.43 | 3.91 ± 3.02 | -0.41 ± 2.00 | 0.67 ± 0.15 | F | E |
| Granivore | Common Waxbill (*Estrilda astrild*) | 1.14 ± 0.15 | -0.44 ± 0.36 | -1.06 ± 0.31 | 0.82 ± 0.26 | 7.30 ± 2.65 | 1.91 ± 1.72 | -0.14 ± 0.11 | H | D |
| Granivore | Cuckoo Finch (*Anomalospiza imberbis*) | -0.62 ± 0.65 | 0.40 ± 1.25 | -1.38 ± 1.36 | -1.84 ± 1.53 | 14.21 ± 8.19 | 11.38 ± 5.97 | -1.20 ± 0.53 | E | E |
| Granivore | Cut-throat Finch (*Amadina fasciata*) | -3.65 ± 0.52 | -0.41 ± 0.75 | 2.82 ± 0.56 | -0.34 ± 0.92 | 3.78 ± 4.35 | 4.08 ± 3.85 | 3.25 ± 0.46 | D | H |
| Granivore | Dusky Indogobird (*Vidua funerea*) | -4.51 ± 2.16 | -1.19 ± 2.12 | -1.68 ± 2.53 | 0.44 ± 2.49 | 15.61 ± 11.69 | -48.03 ± 40.99 | 3.97 ± 1.81 | G | D |
| Granivore | Emerald-spotted Wood-Dove (*Turtur chalcospilos*) | -5.45 ± 1.66 | -1.98 ± 0.66 | -17.36 ± 4.19 | -4.04 ± 1.05 | 22.78 ± 11.49 | 13.59 ± 3.29 | 6.71 ± 1.66 | D | D |
| Granivore | Fan-tailed Widowbird (*Euplectes axillaris*) | -1.53 ± 0.38 | -1.23 ± 2.49 | -1.46 ± 1.04 | 3.57 ± 0.64 | 4.05 ± 20.70 | 7.73 ± 7.59 | -2.21 ± 0.45 | H | D |
| Granivore | Golden-breasted Bunting (*Emberiza flaviventris*) | -2.70 ± 0.47 | 0.21 ± 0.28 | -3.31 ± 0.83 | -2.06 ± 0.56 | -0.45 ± 3.81 | -3.51 ± 2.23 | 4.43 ± 0.46 | F | F |
| Granivore | Great Sparrow (*Passer motitensis*) | -5.87 ± 1.02 | 1.69 ± 0.54 | -0.27 ± 1.29 | 2.82 ± 0.79 | -8.95 ± 9.06 | -14.82 ± 5.92 | 5.11 ± 0.98 | B | F |
| Granivore | Green-winged Pytilia (*Pytilia melba*) | -1.23 ± 0.28 | -0.82 ± 0.59 | -1.15 ± 0.64 | 0.64 ± 0.50 | 3.21 ± 4.94 | -3.33 ± 3.40 | 1.83 ± 0.23 | G | D |
| Granivore | House Sparrow (*Passer domesticus*) | 1.09 ± 0.13 | -0.88 ± 0.41 | 1.97 ± 0.19 | 0.43 ± 0.23 | 2.76 ± 2.40 | -2.10 ± 2.15 | -0.18 ± 0.09 | G | H |
| Granivore | Jameson's Firefinch (*Lagonosticta rhodopareia*) | -0.54 ± 0.27 | -0.65 ± 0.42 | -0.15 ± 0.47 | -1.81 ± 0.56 | 1.96 ± 3.24 | 5.74 ± 2.28 | 1.80 ± 0.22 | D | D |
| Granivore | Laughing Dove (*Spilopelia senegalensis*) | 2.03 ± 0.11 | -0.54 ± 0.22 | 0.55 ± 0.16 | 0.55 ± 0.17 | 1.02 ± 1.76 | -0.70 ± 1.18 | 0.05 ± 0.07 | G | G |
| Granivore | Long-tailed Paradise-Whydah (*Vidua paradisaea*) | -1.05 ± 0.34 | -0.10 ± 0.55 | -2.15 ± 0.88 | 0.83 ± 0.60 | -6.94 ± 8.51 | -6.62 ± 4.32 | 1.52 ± 0.26 | G | C |
| Granivore | Long-tailed Widowbird (*Euplectes pr*ogne) | 2.40 ± 0.11 | -0.79 ± 0.49 | -3.44 ± 0.32 | 0.25 ± 0.20 | 9.88 ± 3.98 | 0.34 ± 2.18 | -2.30 ± 0.12 | G | D |
| Granivore | Namaqua Dove (*Oena capensis*) | -0.04 ± 0.18 | -0.71 ± 0.44 | -4.08 ± 0.69 | 1.77 ± 0.30 | 9.95 ± 3.84 | -2.91 ± 2.37 | 0.90 ± 0.14 | G | D |
| Granivore | Orange-breasted Waxbill (*Sporaeginthus subflavus*) | 0.12 ± 0.27 | -1.27 ± 0.98 | -0.15 ± 0.51 | 0.86 ± 0.51 | 6.96 ± 6.32 | 4.24 ± 4.13 | -0.77 ± 0.23 | H | D |
| Granivore | Pink-billed Lark (*Spizocorys conirostris*) | 0.33 ± 0.61 | -193.64 ± 58.73 | 11.21 ± 4.18 | -1.53 ± 1.12 | -5.96 ± 294.32 | -59.93 ± 152.61 | -4.06 ± 1.18 | C | C |
| Granivore | Pin-tailed Whydah (*Vidua macroura*) | 1.64 ± 0.15 | -0.35 ± 0.31 | -1.27 ± 0.27 | 0.79 ± 0.22 | 5.05 ± 2.54 | -0.86 ± 1.64 | -0.33 ± 0.09 | G | D |
| Granivore | Red-billed Firefinch (*Lagonosticta senegala*) | -1.63 ± 0.35 | -1.55 ± 0.65 | -2.06 ± 0.84 | 0.15 ± 0.56 | -4.33 ± 7.03 | 4.47 ± 3.19 | 2.59 ± 0.29 | H | C |
| Granivore | Red-billed Quelea (*Quelea quelea*) | 1.80 ± 0.11 | -1.00 ± 0.31 | -3.05 ± 0.32 | 1.08 ± 0.17 | 2.45 ± 3.11 | -0.12 ± 1.50 | 0.05 ± 0.08 | G | D |
| Granivore | Red-capped Lark (*Calandrella cinerea*) | 1.07 ± 0.20 | -0.57 ± 0.90 | -5.75 ± 0.81 | 1.64 ± 0.33 | -0.73 ± 12.15 | -5.36 ± 4.18 | -1.97 ± 0.21 | G | C |
| Granivore | Red-collared Widowbird (*Euplectes ardens*) | 1.47 ± 0.17 | -0.03 ± 0.38 | -0.74 ± 0.36 | -2.26 ± 0.38 | 6.16 ± 2.91 | 4.90 ± 2.07 | -0.92 ± 0.14 | D | D |
| Granivore | Red-eyed Dove (*Streptopelia semitorquata*) | 1.83 ± 0.11 | -0.39 ± 0.27 | 1.02 ± 0.18 | -0.01 ± 0.18 | -2.10 ± 2.28 | 4.35 ± 1.34 | -0.57 ± 0.08 | D | G |
| Granivore | Red-headed Finch (*Amadina erythrocephala*) | 0.27 ± 0.22 | -1.27 ± 0.97 | 2.53 ± 0.32 | -0.56 ± 0.46 | -0.18 ± 5.56 | -3.36 ± 5.52 | -0.95 ± 0.18 | C | G |
| Granivore | Scaly-feathered Finch (*Sporopipes squamifrons*) | -2.69 ± 0.30 | -1.06 ± 0.63 | 1.29 ± 0.49 | 0.97 ± 0.46 | -3.23 ± 5.17 | -5.29 ± 3.94 | 3.00 ± 0.26 | G | G |
| Granivore | Shaft-tailed Whydah (*Vidua regia*) | -3.90 ± 0.63 | -0.97 ± 0.93 | -2.93 ± 1.47 | 1.94 ± 0.72 | 11.71 ± 6.84 | -10.85 ± 6.14 | 3.76 ± 0.57 | G | D |
| Granivore | Southern Red Bishop (*Euplectes orix*) | 2.07 ± 0.12 | -0.95 ± 0.33 | 0.27 ± 0.18 | 0.46 ± 0.18 | 1.71 ± 2.45 | 3.09 ± 1.55 | -0.92 ± 0.08 | H | G |
| Granivore | Speckled Pigeon (*Columba guinea*) | 1.44 ± 0.13 | -0.29 ± 0.29 | 0.36 ± 0.20 | 0.72 ± 0.20 | 0.10 ± 2.42 | -1.26 ± 1.56 | -0.33 ± 0.08 | G | G |
| Granivore | Village Indigobird (*Vidua chalybeata*) | -1.89 ± 0.45 | -1.98 ± 1.13 | -2.33 ± 1.14 | 0.01 ± 0.80 | 3.24 ± 9.18 | -10.02 ± 7.87 | 2.12 ± 0.38 | G | C |
| Granivore | Violet-eared Waxbill (*Granatina granatina*) | -1.64 ± 0.39 | 0.51 ± 0.37 | -1.75 ± 0.82 | -0.81 ± 0.68 | -3.63 ± 4.99 | -11.79 ± 4.06 | 2.62 ± 0.34 | F | F |
| Granivore | White-browed Sparrow-Weaver (*Plocepasser mahali*) | 1.71 ± 0.13 | -2.55 ± 0.55 | -3.79 ± 0.44 | -0.81 ± 0.25 | 8.86 ± 4.28 | 6.84 ± 2.38 | -0.65 ± 0.11 | D | D |
| Granivore | White-winged Widowbird (*Euplectes albonotatus*) | 0.70 ± 0.14 | -0.35 ± 0.31 | -1.68 ± 0.35 | 1.63 ± 0.21 | 4.35 ± 2.78 | 0.77 ± 1.50 | 0.57 ± 0.09 | H | D |
| Granivore | Yellow Bishop (*Euplectes capensis*) | -2.35 ± 1.15 | 3.93 ± 2.25 | 0.22 ± 2.17 | -0.11 ± 2.47 | -15.76 ± 31.08 | 6.45 ± 9.07 | -3.18 ± 1.54 | E | B |
| Granivore | Yellow Canary (*Crithagra flaviventris*) | 0.76 ± 0.20 | -0.94 ± 0.68 | -2.49 ± 0.55 | 0.51 ± 0.37 | -10.42 ± 9.19 | -1.77 ± 3.57 | -0.68 ± 0.17 | G | C |
| Granivore | Yellow-crowned Bishop (*Euplectes afer*) | 1.31 ± 0.15 | -0.51 ± 0.49 | -2.04 ± 0.36 | 1.62 ± 0.25 | 4.28 ± 4.10 | -6.18 ± 2.60 | -0.75 ± 0.12 | G | D |
| Granivore | Yellow-fronted Canary (*Crithagra mozambicus*) | 0.50 ± 0.18 | -0.14 ± 0.25 | -0.57 ± 0.35 | -2.14 ± 0.37 | -1.38 ± 2.52 | 4.62 ± 1.50 | 1.33 ± 0.14 | D | C |
| Granivore | Yellow-throated Petronia (*Petronia superciliaris*) | -1.74 ± 0.55 | 0.35 ± 0.46 | -2.10 ± 1.15 | -3.27 ± 1.12 | -6.25 ± 6.51 | -1.98 ± 3.94 | 2.57 ± 0.50 | F | F |
| GF | African Grey Hornbill (*Tockus nasutus*) | -0.46 ± 0.21 | -0.45 ± 0.26 | -0.59 ± 0.39 | -2.69 ± 0.41 | -2.13 ± 2.64 | 7.37 ± 1.47 | 2.41 ± 0.18 | D | C |
| GF | African Hoopoe (*Upupa africana*) | 0.81 ± 0.16 | 0.10 ± 0.35 | 1.69 ± 0.26 | -1.55 ± 0.34 | 1.23 ± 2.43 | 1.49 ± 2.05 | -0.09 ± 0.12 | E | A |
| GF | African Pipit (*Anthus cinnamomeus*) | 2.12 ± 0.11 | 0.08 ± 0.26 | -2.28 ± 0.27 | 0.62 ± 0.18 | 5.89 ± 2.28 | -5.22 ± 1.58 | -0.74 ± 0.09 | B | E |
| GF | African Sacred Ibis (*Threskiornis aethiopicus*) | 0.89 ± 0.13 | 0.23 ± 0.41 | 2.45 ± 0.20 | 0.21 ± 0.26 | 2.24 ± 2.67 | 0.55 ± 2.14 | -0.98 ± 0.11 | A | A |
| GF | African Stonechat (*Saxicola torquatus*) | 2.29 ± 0.10 | -0.45 ± 0.34 | -1.77 ± 0.24 | 0.47 ± 0.18 | 9.62 ± 2.52 | 1.81 ± 1.56 | -1.59 ± 0.09 | H | D |
| GF | Anteating Chat (*Myrmecocichla formicivora*) | 1.69 ± 0.16 | -0.41 ± 0.60 | -6.78 ± 0.81 | -0.74 ± 0.33 | 12.86 ± 5.85 | 1.41 ± 2.98 | -2.01 ± 0.17 | D | D |
| GF | Arrow-marked Babbler (*Turdoides jardineii*) | -0.32 ± 0.19 | -0.12 ± 0.22 | -1.64 ± 0.41 | -1.29 ± 0.33 | -0.33 ± 2.42 | 4.48 ± 1.20 | 2.47 ± 0.16 | D | C |
| GF | Black-crowned Tchagra (*Tchagra* senegalus) | -0.57 ± 0.28 | 0.73 ± 0.31 | -1.78 ± 0.68 | -1.68 ± 0.58 | 2.85 ± 3.39 | 2.31 ± 1.96 | 1.47 ± 0.23 | E | E |
| GF | Black-headed Heron (*Ardea melanocephala*) | 1.29 ± 0.13 | -1.43 ± 0.50 | 0.78 ± 0.21 | 1.72 ± 0.20 | 2.94 ± 3.30 | 4.52 ± 1.96 | -0.85 ± 0.09 | H | H |
| GF | Blue Korhaan (*Eupodotis caerulescens*) | -0.07 ± 0.51 | -53.27 ± 82.41 | -18.52 ± 6.75 | -1.06 ± 0.93 | 0.09 ± 147.30 | -15.55 ± 223.89 | -3.36 ± 0.87 | C | C |
| GF | Bokmakierie Bokmakierie (*Telophorus zeylonus*) | 2.09 ± 0.14 | -0.74 ± 0.44 | -2.64 ± 0.39 | -1.73 ± 0.30 | 3.82 ± 3.98 | 4.26 ± 2.32 | -1.40 ± 0.13 | D | D |
| GF | Brown-crowned Tchagra (*Tchagra australis*) | 0.29 ± 0.19 | -0.58 ± 0.26 | -1.51 ± 0.40 | -1.70 ± 0.37 | -2.88 ± 2.92 | 0.33 ± 1.76 | 2.05 ± 0.16 | C | C |
| GF | Brown-hooded Kingfisher (*Halcyon albiventris*) | -0.34 ± 0.20 | -0.71 ± 0.29 | 0.19 ± 0.33 | -1.62 ± 0.38 | 4.66 ± 2.12 | 6.67 ± 1.54 | 2.18 ± 0.16 | D | H |
| GF | Buffy Pipit (*Anthus vaalensis*) | 0.74 ± 0.41 | 1.15 ± 0.50 | -3.51 ± 1.09 | -3.13 ± 0.98 | 9.50 ± 4.74 | -2.89 ± 4.18 | -0.54 ± 0.32 | F | E |
| GF | Bushveld Pipit (*Anthus caffer*) | -5.07 ± 2.19 | -0.57 ± 0.77 | -7.75 ± 4.02 | -4.78 ± 2.01 | 4.80 ± 13.26 | -8.49 ± 10.36 | 5.34 ± 2.18 | C | D |
| GF | Cape Crow (*Corvus capensis*) | -1.56 ± 0.46 | -0.55 ± 1.47 | -5.91 ± 2.35 | 1.90 ± 0.80 | -20.72 ± 35.97 | -3.86 ± 7.71 | -0.15 ± 0.39 | G | C |
| GF | Cape Grassbird (*Sphenoeacus afer*) | 0.06 ± 0.29 | 0.63 ± 0.48 | -3.08 ± 0.91 | -1.32 ± 0.61 | 10.86 ± 4.45 | 8.42 ± 2.27 | -0.46 ± 0.24 | E | E |
| GF | Cape Longclaw (*Macronyx capensis*) | 2.38 ± 0.11 | -0.37 ± 0.40 | -3.08 ± 0.30 | 0.12 ± 0.20 | 12.66 ± 2.97 | -0.58 ± 1.94 | -2.01 ± 0.11 | G | D |
| GF | Cape Robin-Chat (*Cossypha caffra*) | 1.33 ± 0.13 | -0.03 ± 0.34 | 2.00 ± 0.21 | -1.09 ± 0.26 | -1.85 ± 2.59 | 7.03 ± 1.66 | -0.91 ± 0.10 | D | G |
| GF | Cape Rock-Thrush (*Monticola rupestris*) | -1.23 ± 0.60 | 3.04 ± 0.80 | 0.89 ± 1.24 | -5.33 ± 2.02 | 8.08 ± 5.49 | 12.69 ± 3.78 | -1.61 ± 0.53 | E | A |
| GF | Cape Wagtail (*Motacilla capensis*) | 1.22 ± 0.13 | 0.13 ± 0.32 | 0.62 ± 0.23 | 0.20 ± 0.23 | 3.90 ± 2.43 | 0.19 ± 1.74 | -0.70 ± 0.10 | A | A |
| GF | Capped Wheatear (*Oenanthe pileata*) | 1.50 ± 0.18 | 1.28 ± 0.46 | -2.23 ± 0.45 | -0.39 ± 0.34 | 1.52 ± 4.88 | -4.95 ± 3.00 | -2.17 ± 0.19 | F | E |
| GF | Cattle Egret (*Bubulcus ibis*) | 1.50 ± 0.12 | -0.71 ± 0.26 | -0.18 ± 0.21 | 0.66 ± 0.18 | 0.33 ± 2.17 | 0.32 ± 1.37 | 0.24 ± 0.08 | G | C |
| GF | Cloud Cisticola (*Cisticola textrix*) | 1.61 ± 0.17 | -0.48 ± 0.59 | -3.89 ± 0.53 | 0.00 ± 0.32 | 4.34 ± 6.09 | -1.31 ± 3.12 | -1.71 ± 0.17 | G | D |
| GF | Common Fiscal (*Lanius collaris*) | 2.41 ± 0.09 | -0.65 ± 0.28 | -0.31 ± 0.18 | 0.17 ± 0.17 | 2.21 ± 2.27 | 3.42 ± 1.34 | -1.15 ± 0.08 | H | D |
| GF | Crowned Lapwing (*Vanellus coronatus*) | 1.75 ± 0.15 | -1.00 ± 0.30 | 0.19 ± 0.19 | 0.30 ± 0.18 | 1.34 ± 2.32 | 3.53 ± 1.47 | -0.33 ± 0.08 | H | G |
| GF | Familiar Chat (*Cercomela familiaris*) | 0.86 ± 0.24 | 1.38 ± 0.30 | -1.63 ± 0.56 | -3.18 ± 0.59 | 5.88 ± 2.89 | -0.56 ± 2.19 | -0.33 ± 0.20 | E | E |
| GF | Fiscal Flycatcher (*Sigelus silens*) | 1.85 ± 0.14 | -0.62 ± 0.39 | 0.18 ± 0.26 | -2.05 ± 0.30 | 2.72 ± 2.84 | 3.86 ± 2.09 | -0.96 ± 0.12 | D | H |
| GF | Greater Kestrel (*Falco rupicoloides*) | 0.24 ± 0.31 | -1.77 ± 1.17 | -3.65 ± 0.93 | 0.25 ± 0.59 | 12.60 ± 7.88 | -3.62 ± 6.23 | -0.67 ± 0.27 | G | D |
| GF | Groundscraper Thrush (*Psophocichla litsipsirupa*) | -0.53 ± 0.23 | -0.12 ± 0.30 | -0.26 ± 0.43 | -1.39 ± 0.44 | -3.28 ± 3.10 | 4.28 ± 1.71 | 2.05 ± 0.19 | D | C |
| GF | Hadeda Ibis (*Bostrychia hagedash*) | 1.77 ± 0.11 | -0.18 ± 0.27 | 1.46 ± 0.17 | 0.24 ± 0.18 | -1.47 ± 2.20 | 1.34 ± 1.45 | -0.70 ± 0.08 | H | G |
| GF | Kurrichane Thrush (*Turdus libonyana*) | -1.29 ± 0.79 | 0.75 ± 0.90 | 0.18 ± 1.32 | -1.47 ± 1.72 | -41.18 ± 29.89 | -0.85 ± 7.58 | 0.75 ± 0.59 | E | B |
| GF | Kurrichane Thrush (*Turdus libonyanus*) | -0.54 ± 0.23 | -0.23 ± 0.32 | 0.49 ± 0.40 | -1.89 ± 0.49 | -0.34 ± 2.81 | 8.11 ± 1.73 | 1.77 ± 0.19 | D | G |
| GF | Lazy Cisticola (*Cisticola aberrans*) | -0.60 ± 0.44 | 0.83 ± 0.52 | -0.46 ± 0.88 | -3.41 ± 1.14 | 5.60 ± 4.44 | 9.94 ± 2.89 | 0.40 ± 0.35 | E | E |
| GF | Lilac-breasted Roller (*Coracias caudatus*) | -2.54 ± 0.41 | -0.63 ± 0.34 | -2.49 ± 0.70 | -2.00 ± 0.52 | -0.22 ± 3.90 | -1.85 ± 2.46 | 4.29 ± 0.40 | C | C |
| GF | Long-billed Pipit (*Anthus similis*) | 0.60 ± 0.37 | 0.19 ± 0.62 | -1.60 ± 0.84 | -4.09 ± 1.01 | 5.56 ± 5.43 | 9.87 ± 3.47 | -0.76 ± 0.31 | E | E |
| GF | Magpie Shrike (*Corvinella melanoleuca*) | -7.98 ± 1.33 | -1.24 ± 0.43 | -1.71 ± 0.60 | -0.41 ± 0.41 | 0.32 ± 4.05 | -1.10 ± 2.57 | 9.37 ± 1.34 | C | C |
| GF | Marico Flycatcher (*Bradornis mariquensis*) | -4.72 ± 0.60 | 0.79 ± 0.29 | -0.28 ± 0.57 | 0.35 ± 0.46 | -1.43 ± 3.25 | -13.86 ± 3.21 | 5.66 ± 0.60 | B | E |
| GF | Mocking Cliff-Chat (*Thamnolaea cinnamomeiventris*) | 0.21 ± 0.35 | -0.05 ± 0.48 | -2.54 ± 0.93 | -4.62 ± 0.93 | 7.56 ± 4.33 | 8.23 ± 2.88 | 0.25 ± 0.29 | D | D |
| GF | Mountain Wheatear (*Oenanthe monticola*) | 1.05 ± 0.22 | 0.33 ± 0.63 | -0.44 ± 0.48 | -2.51 ± 0.53 | -8.27 ± 7.28 | 9.03 ± 3.03 | -1.94 ± 0.23 | E | F |
| GF | Neddicky Neddicky (*Cisticola fulvicapilla*) | 1.52 ± 0.13 | -0.62 ± 0.26 | -1.80 ± 0.31 | -1.41 ± 0.25 | -1.23 ± 2.74 | 2.59 ± 1.46 | 0.44 ± 0.10 | D | C |
| GF | Pearl-spotted Owlet (*Glaucidium perlatum*) | -3.64 ± 0.72 | -1.13 ± 0.51 | -4.72 ± 1.31 | -1.68 ± 0.72 | 12.29 ± 4.61 | 4.36 ± 2.85 | 4.83 ± 0.70 | D | D |
| GF | Pied Crow (*Corvus albus*) | 1.20 ± 0.14 | -0.23 ± 0.26 | 0.93 ± 0.22 | -1.50 ± 0.26 | -1.32 ± 2.23 | -2.31 ± 1.74 | 0.39 ± 0.10 | C | G |
| GF | Pied Starling (*Spreo bicolor*) | 1.22 ± 0.18 | -1.37 ± 0.87 | -1.07 ± 0.41 | -0.79 ± 0.37 | 13.92 ± 4.89 | 4.80 ± 3.82 | -2.09 ± 0.20 | D | D |
| GF | Plain-backed Pipit (*Anthus leucophrys*) | 0.67 ± 0.43 | 1.44 ± 0.56 | -2.91 ± 1.04 | -2.81 ± 1.01 | 10.71 ± 4.80 | -2.86 ± 4.40 | -1.12 ± 0.35 | E | E |
| GF | Red-throated Wryneck (*Jynx ruficollis*) | 1.69 ± 0.20 | -0.88 ± 0.70 | -1.57 ± 0.44 | -2.17 ± 0.44 | 10.52 ± 4.36 | 4.87 ± 3.45 | -1.86 ± 0.19 | D | D |
| GF | Rufous-naped Lark (*Mirafra africana*) | 2.19 ± 0.13 | 0.05 ± 0.23 | -2.57 ± 0.31 | -1.30 ± 0.24 | 4.83 ± 2.29 | -2.39 ± 1.52 | -0.31 ± 0.10 | F | E |
| GF | Secretarybird Secretarybird (*Sagittarius serpentarius*) | -0.04 ± 0.41 | 0.72 ± 0.89 | -12.90 ± 3.48 | 0.54 ± 0.71 | 12.11 ± 17.37 | -5.91 ± 5.88 | -1.45 ± 0.39 | B | E |
| GF | Sentinel Rock-Thrush (*Monticola explorator*) | -1.18 ± 0.84 | 2.06 ± 1.38 | -3.77 ± 3.00 | -4.00 ± 2.32 | 0.55 ± 17.62 | 8.83 ± 6.96 | -2.02 ± 0.87 | E | E |
| GF | Southern Black Flycatcher (*Melaenornis pammelaina*) | -0.70 ± 0.30 | -0.03 ± 0.32 | -2.76 ± 0.79 | -2.64 ± 0.61 | 2.88 ± 3.71 | 5.87 ± 1.94 | 2.16 ± 0.26 | D | D |
| GF | Southern White-crowned Shrike (*Eurocephalus anguitimens*) | -9.55 ± 4.65 | -1.42 ± 1.18 | -1.96 ± 1.94 | -2.38 ± 1.65 | 1.22 ± 10.39 | 6.86 ± 6.81 | 9.46 ± 4.67 | D | C |
| GF | Southern Yellow-billed Hornbill (*Tockus leucomelas*) | -7.80 ± 1.73 | -0.75 ± 0.39 | -3.76 ± 0.93 | -2.70 ± 0.61 | 6.42 ± 3.98 | -1.52 ± 2.93 | 9.16 ± 1.74 | C | D |
| GF | Spike-heeled Lark (*Chersomanes albofasciata*) | 1.09 ± 0.24 | -1.69 ± 1.18 | -3.46 ± 0.77 | -1.71 ± 0.53 | 15.82 ± 7.74 | 11.20 ± 4.97 | -2.43 ± 0.28 | D | D |
| GF | Spotted Eagle-Owl (*Bubo africanus*) | 0.13 ± 0.33 | 0.07 ± 0.72 | 1.41 ± 0.54 | -1.98 ± 0.78 | -10.86 ± 7.45 | 9.38 ± 3.76 | -0.59 ± 0.27 | E | B |
| GF | Spotted Thick-knee (*Burhinus capensis*) | 0.94 ± 0.17 | 0.10 ± 0.39 | 2.22 ± 0.23 | -0.46 ± 0.32 | -0.37 ± 2.80 | 1.32 ± 2.17 | -0.57 ± 0.12 | E | B |
| GF | Striped Kingfisher (*Halcyon chelicuti*) | -2.61 ± 0.84 | -0.94 ± 0.72 | -4.57 ± 2.17 | -4.35 ± 1.44 | 0.05 ± 10.48 | -2.21 ± 6.62 | 3.15 ± 0.80 | C | C |
| GF | Striped Pipit (*Anthus lineiventris*) | -0.84 ± 0.52 | 0.00 ± 0.67 | -0.79 ± 1.13 | -4.90 ± 1.43 | -1.77 ± 7.29 | 11.48 ± 3.88 | 0.64 ± 0.44 | D | C |
| GF | Wailing Cisticola (*Cisticola lais*) | 0.57 ± 0.26 | 0.15 ± 0.61 | -1.89 ± 0.71 | -2.15 ± 0.61 | -0.08 ± 6.63 | 7.69 ± 3.10 | -1.14 ± 0.24 | E | E |
| GF | Wattled Starling (*Creatophora cinerea*) | 0.85 ± 0.19 | -2.32 ± 0.86 | -0.35 ± 0.38 | 0.12 ± 0.36 | -1.34 ± 6.27 | 2.92 ± 3.93 | -0.50 ± 0.15 | G | C |
| GF | White Stork (*Ciconia ciconia*) | -0.50 ± 0.32 | 0.34 ± 0.67 | -0.15 ± 0.61 | 1.06 ± 0.61 | 0.53 ± 5.96 | 2.55 ± 3.30 | 0.15 ± 0.24 | A | E |
| GF | White-browed Scrub-Robin (*Cercotrichas leucophrys*) | -2.98 ± 0.37 | -0.12 ± 0.26 | -1.55 ± 0.51 | -0.44 ± 0.38 | 0.22 ± 2.87 | -0.40 ± 1.64 | 4.85 ± 0.36 | C | C |
| GF | White-throated Robin-Chat (*Cossypha humeralis*) | -3.97 ± 0.58 | 0.24 ± 0.34 | -0.48 ± 0.59 | -0.32 ± 0.54 | -1.05 ± 3.52 | 0.55 ± 2.06 | 5.19 ± 0.57 | E | F |
| Hawker | African Palm-Swift (*Cypsiurus parvus*) | 0.97 ± 0.13 | -0.40 ± 0.30 | 1.91 ± 0.20 | -0.64 ± 0.26 | 0.43 ± 2.17 | 4.01 ± 1.59 | 0.18 ± 0.10 | D | G |
| Hawker | Alpine Swift (*Tachymarptis melba*) | -1.12 ± 0.77 | 1.20 ± 0.77 | -1.51 ± 1.50 | -1.41 ± 1.68 | 3.94 ± 8.28 | 3.46 ± 4.94 | 0.81 ± 0.56 | E | E |
| Hawker | Common House-Martin (*Delichon urbicum*) | 0.42 ± 0.29 | -0.42 ± 0.54 | -1.20 ± 0.57 | 0.54 ± 0.51 | -12.53 ± 7.78 | 3.20 ± 2.87 | 0.42 ± 0.20 | H | C |
| Hawker | Fiery-necked Nightjar (*Caprimulgus pectoralis*) | -1.61 ± 0.57 | 0.28 ± 0.50 | -0.14 ± 0.95 | -3.43 ± 1.23 | -15.18 ± 7.57 | 4.70 ± 3.65 | 2.56 ± 0.51 | E | F |
| Hawker | Fork-tailed Drongo (*Dicrurus adsimilis*) | -0.81 ± 0.19 | -0.23 ± 0.20 | -1.21 ± 0.36 | -0.85 ± 0.30 | 1.98 ± 2.02 | 1.82 ± 1.25 | 3.18 ± 0.16 | D | D |
| Hawker | Lesser Striped Swallow (*Hirundo abyssinica*) | -0.17 ± 0.19 | 0.20 ± 0.23 | 0.40 ± 0.31 | -0.78 ± 0.34 | -7.71 ± 2.69 | 2.61 ± 1.33 | 2.11 ± 0.15 | E | B |
| Hawker | Little Bee-eater (*Merops pusillus*) | -0.61 ± 0.48 | -0.09 ± 0.66 | -2.17 ± 1.07 | -0.84 ± 1.02 | 4.11 ± 5.96 | 3.70 ± 3.88 | 0.91 ± 0.36 | D | D |
| Hawker | Little Swift (*Apus affinis*) | 0.87 ± 0.15 | -0.14 ± 0.33 | 0.86 ± 0.25 | 0.10 ± 0.27 | -4.13 ± 2.98 | -2.06 ± 1.97 | 0.15 ± 0.11 | G | G |
| Hawker | Pearl-breasted Swallow (*Hirundo dimidiata*) | -0.44 ± 0.29 | 0.42 ± 0.30 | -1.53 ± 0.60 | -1.37 ± 0.54 | -0.38 ± 3.33 | -3.26 ± 2.30 | 1.88 ± 0.23 | F | E |
| Hawker | Rock Martin (*Hirundo fuligula*) | 0.05 ± 0.22 | 0.63 ± 0.44 | 3.09 ± 0.31 | -0.87 ± 0.50 | -1.38 ± 3.23 | 6.89 ± 2.20 | -0.26 ± 0.17 | E | B |
| Hawker | White-fronted Bee-eater (*Merops bullockoides*) | -0.40 ± 0.21 | -1.21 ± 0.45 | -0.19 ± 0.44 | -0.87 ± 0.43 | 3.00 ± 3.32 | 7.56 ± 2.17 | 1.46 ± 0.17 | D | D |
| Predator | African Grass-Owl (*Tyto capensis*) | -0.68 ± 0.82 | 0.30 ± 3.02 | -2.96 ± 2.12 | -1.62 ± 1.87 | 8.91 ± 23.59 | 3.40 ± 15.14 | -3.09 ± 1.09 | E | E |
| Predator | African Harrier-Hawk (*Polyboroides typus*) | -1.16 ± 0.47 | 1.43 ± 0.64 | 2.63 ± 0.62 | 0.09 ± 1.03 | -0.17 ± 5.00 | 6.08 ± 3.30 | 0.45 ± 0.34 | A | A |
| Predator | African Hawk-Eagle (*Aquila spilogaster*) | -1.91 ± 0.89 | 0.30 ± 0.85 | -2.66 ± 2.13 | -3.07 ± 2.07 | 2.16 ± 9.07 | 5.55 ± 5.73 | 1.76 ± 0.76 | E | E |
| Predator | Barn Owl (*Tyto alba*) | 0.10 ± 0.33 | 0.90 ± 0.45 | -0.92 ± 0.68 | -0.97 ± 0.70 | -15.15 ± 8.53 | -6.80 ± 4.23 | 0.20 ± 0.26 | F | F |
| Predator | Black Sparrowhawk (*Accipiter melanoleucus*) | -0.26 ± 0.40 | -0.60 ± 1.14 | 2.36 ± 0.56 | -0.80 ± 0.89 | 0.44 ± 7.34 | 9.23 ± 5.36 | -0.86 ± 0.32 | D | G |
| Predator | Black-chested Snake-Eagle (*Circaetus pectoralis*) | 0.19 ± 0.31 | -0.17 ± 0.44 | -4.15 ± 0.94 | -0.98 ± 0.58 | 3.06 ± 5.06 | 2.89 ± 2.55 | 0.84 ± 0.23 | D | D |
| Predator | Black-shouldered Kite (*Elanus caeruleus*) | 1.37 ± 0.15 | -0.10 ± 0.29 | -0.75 ± 0.25 | 1.42 ± 0.20 | 2.70 ± 2.64 | -0.37 ± 1.49 | -0.36 ± 0.09 | G | D |
| Predator | Brown Snake-Eagle (*Circaetus cinereus*) | -0.90 ± 0.55 | -0.69 ± 0.72 | -9.00 ± 2.89 | -1.73 ± 1.10 | 16.19 ± 8.16 | 7.56 ± 3.78 | 1.58 ± 0.45 | D | D |
| Predator | Gabar Goshawk (*Melierax gabar*) | -0.58 ± 0.40 | 0.14 ± 0.44 | -2.31 ± 0.86 | -1.13 ± 0.76 | 0.82 ± 4.76 | -4.98 ± 3.74 | 1.77 ± 0.32 | F | E |
| Predator | Hamerkop Hamerkop (*Scopus umbretta*) | 0.65 ± 0.21 | -0.87 ± 0.43 | -0.88 ± 0.41 | -0.34 ± 0.38 | -6.02 ± 4.40 | 3.10 ± 2.25 | 0.85 ± 0.15 | D | C |
| Predator | Jackal Buzzard (*Buteo rufofuscus*) | 0.31 ± 0.48 | -0.12 ± 1.16 | -6.01 ± 1.93 | -2.40 ± 1.13 | 28.58 ± 7.06 | 4.00 ± 6.33 | -1.66 ± 0.43 | D | D |
| Predator | Lanner Falcon (*Falco biarmicus*) | 0.64 ± 0.46 | -1.24 ± 1.00 | -3.78 ± 1.10 | -0.96 ± 0.86 | 12.18 ± 7.05 | 5.48 ± 4.95 | -0.42 ± 0.33 | D | D |
| Predator | Little Sparrowhawk (*Accipiter minullus*) | 0.60 ± 0.37 | -0.57 ± 0.66 | 1.22 ± 0.54 | -4.38 ± 0.98 | -11.19 ± 6.78 | 10.55 ± 3.86 | -0.07 ± 0.28 | D | G |
| Predator | Marsh Owl (*Asio* capensis) | 0.38 ± 0.29 | -0.79 ± 1.02 | -2.33 ± 0.68 | 1.38 ± 0.53 | 9.98 ± 7.34 | 1.48 ± 4.50 | -1.28 ± 0.26 | G | D |
| Predator | Martial Eagle (*Polemaetus bellicosus*) | 2.15 ± 1.15 | -0.30 ± 1.06 | -12.79 ± 7.63 | -6.31 ± 3.14 | -6.85 ± 38.78 | -46.92 ± 38.01 | 0.21 ± 0.99 | C | C |
| Predator | Rock Kestrel (*Falco rupicolus*) | 0.16 ± 0.36 | 1.35 ± 0.54 | -4.11 ± 1.17 | -0.10 ± 0.68 | -5.40 ± 9.64 | 2.80 ± 2.99 | -0.96 ± 0.30 | E | F |
| Predator | Shikra Shikra (*Accipiter badius*) | -1.08 ± 0.92 | -0.96 ± 0.84 | -1.17 ± 1.22 | -2.45 ± 1.46 | -21.60 ± 15.15 | 1.46 ± 6.61 | 2.77 ± 0.65 | C | C |
| Predator | Tawny Eagle (*Aquila rapax*) | -5.47 ± 3.51 | 0.48 ± 1.54 | -2.61 ± 6.05 | -4.25 ± 4.89 | -32.55 ± 46.74 | 9.41 ± 11.28 | 4.24 ± 3.37 | E | F |
| Predator | Verreaux's Eagle (*Aquila verreauxii*) | -2.13 ± 0.50 | 1.08 ± 0.77 | 3.74 ± 0.68 | -1.61 ± 1.29 | -0.44 ± 5.26 | 9.27 ± 3.89 | 0.58 ± 0.37 | E | A |
| Vegivore | Blue Crane (*Anthropoides paradiseus*) | -1.48 ± 0.61 | 1.00 ± 1.73 | -6.63 ± 3.28 | 0.32 ± 1.18 | 2.47 ± 28.46 | -3.26 ± 10.21 | -1.92 ± 0.69 | B | E |
| Vegivore | Cape Bunting (*Emberiza capensis*) | 0.03 ± 0.41 | 0.64 ± 0.66 | -3.26 ± 1.35 | -4.25 ± 1.11 | 3.44 ± 7.30 | 12.53 ± 3.25 | -0.91 ± 0.36 | E | E |
| Vegivore | Cape Weaver (*Ploceus capensis*) | 0.27 ± 0.27 | 0.18 ± 0.48 | 0.29 ± 0.49 | -1.76 ± 0.62 | 3.96 ± 3.84 | 5.51 ± 2.66 | -0.19 ± 0.22 | E | A |
| Vegivore | Coqui Francolin (*Peliperdix coqui*) | 0.43 ± 0.30 | -0.37 ± 0.38 | -4.13 ± 0.96 | -3.68 ± 0.67 | -3.32 ± 5.72 | 2.26 ± 2.80 | 1.06 ± 0.24 | D | C |
| Vegivore | Crested Francolin (*Dendroperdix sephaena*) | -2.96 ± 0.42 | -0.25 ± 0.27 | -3.36 ± 0.71 | -1.42 ± 0.43 | 1.43 ± 3.41 | 1.22 ± 1.72 | 4.78 ± 0.41 | D | D |
| Vegivore | Egyptian Goose (*Alopochen aegyptiaca*) | 1.34 ± 0.12 | 0.93 ± 0.26 | 0.81 ± 0.21 | 0.86 ± 0.20 | -1.29 ± 2.50 | -2.11 ± 1.53 | -0.95 ± 0.09 | B | A |
| Vegivore | Grey Go-away-bird (*Corythaixoides concolor*) | -0.07 ± 0.15 | 0.39 ± 0.24 | 2.25 ± 0.21 | -1.54 ± 0.31 | -7.40 ± 2.29 | 2.71 ± 1.36 | 1.58 ± 0.11 | E | B |
| Vegivore | Helmeted Guineafowl (*Numida meleagris*) | 1.65 ± 0.12 | 0.16 ± 0.24 | -0.71 ± 0.23 | 0.07 ± 0.20 | 1.67 ± 2.25 | -0.21 ± 1.39 | -0.38 ± 0.08 | A | E |
| Vegivore | Natal Spurfowl (*Pternistis natalensis*) | -1.00 ± 0.28 | 0.63 ± 0.27 | -2.94 ± 0.75 | -2.10 ± 0.52 | 5.41 ± 2.98 | 0.32 ± 1.81 | 2.35 ± 0.25 | E | E |
| Vegivore | Orange River Francolin (*Scleroptila levaillantoides*) | 1.44 ± 0.21 | -7.94 ± 2.68 | -4.50 ± 0.70 | -0.51 ± 0.40 | 39.31 ± 11.37 | 5.69 ± 8.21 | -2.78 ± 0.27 | C | D |
| Vegivore | Red-winged Francolin (*Scleroptila levaillantii*) | 0.11 ± 0.55 | 0.22 ± 1.05 | -3.58 ± 1.67 | -4.57 ± 1.56 | 12.73 ± 8.87 | 11.59 ± 5.37 | -1.57 ± 0.48 | E | E |
| Vegivore | Shelley's Francolin (*Scleroptila shelleyi*) | -3.54 ± 1.18 | 0.04 ± 1.85 | -0.05 ± 2.17 | -0.27 ± 2.50 | 10.56 ± 11.14 | 1.80 ± 10.52 | 1.16 ± 0.93 | E | E |
| Vegivore | Southern Masked-Weaver (*Ploceus velatus*) | 2.24 ± 0.13 | -0.56 ± 0.22 | 0.58 ± 0.16 | 0.28 ± 0.17 | -1.84 ± 1.91 | 1.80 ± 1.16 | -0.27 ± 0.07 | H | G |
| Vegivore | Speckled Mousebird (*Colius striatus*) | 0.90 ± 0.14 | -0.46 ± 0.31 | 2.53 ± 0.19 | -0.75 ± 0.26 | -5.70 ± 2.46 | 8.03 ± 1.48 | 0.27 ± 0.10 | D | G |
| Vegivore | Spur-winged Goose (*Plectropterus gambensis*) | 0.87 ± 0.15 | 0.91 ± 0.36 | -1.33 ± 0.37 | 1.52 ± 0.27 | 3.44 ± 3.63 | -1.78 ± 1.95 | -1.01 ± 0.13 | A | E |
| Vegivore | Streaky-headed Seedeater (*Crithagra gularis*) | 0.84 ± 0.18 | -0.30 ± 0.40 | 1.02 ± 0.31 | -2.26 ± 0.41 | -0.78 ± 3.16 | 2.56 ± 2.43 | -0.08 ± 0.14 | D | G |
| Vegivore | Swainson's Spurfowl (*Pternistis swainsonii*) | 1.85 ± 0.12 | -0.34 ± 0.25 | -3.00 ± 0.30 | 0.87 ± 0.18 | 3.17 ± 2.64 | -0.41 ± 1.30 | -0.14 ± 0.08 | G | D |
| Vegivore | Village Weaver (*Ploceus cucullatus*) | -1.23 ± 0.30 | -0.64 ± 0.52 | 1.00 ± 0.47 | 0.29 ± 0.58 | -1.23 ± 4.20 | 0.78 ± 2.90 | 1.98 ± 0.24 | G | G |
| Vegivore | White-bellied Korhaan (*Eupodotis senegalensis*) | -2.30 ± 0.84 | -2.02 ± 2.32 | -2.03 ± 2.34 | -2.67 ± 1.89 | -10.01 ± 30.59 | 7.63 ± 11.77 | 0.72 ± 0.70 | D | C |

**Table A3.** Percentage of species of each guild that fall into the interaction cases (A-H, Fig. 2) based on the protected areas x urban land-use interaction (1), and the protected areas x agricultural land-use interaction (2), but limited to species for which each interaction is significant at the 5% confidence interval. Cases A, B, C, and D indicate situations where the proportion-abundance relationship becomes more positive with increasing agricultural or urban area in the same pentad (termed ‘positive interaction cases’). Cases E, F, G, and H indicate situations where the proportion-abundance relationship becomes more negative within increasing surrounding agricultural or urban area (termed ‘negative interaction cases’). Each row sums to 100%. Column ‘Total’ under ‘Positive Interaction Cases’ heading sums the percentage of cases A-H for each feeding-guild, and likewise, column ‘Total’ under ‘Negative Interaction Cases’ heading sums the percentage of cases E-H.

**Panel 1: Urban**

| Row | Feeding guild | Positive Interaction Cases | | | | | Negative Interaction Cases | | | | |
| --- | --- | --- | --- | --- | --- | --- | --- | --- | --- | --- | --- |
|  |  | A | B | C | D | Total | E | F | G | H | Total |
| 1 | Frugivores | 0 | 67 | 0 | 0 | 67 | 0 | 0 | 33 | 0 | 33 |
| 2 | Gleaners | 0 | 0 | 0 | 50 | 50 | 50 | 0 | 0 | 0 | 50 |
| 3 | Granivores | 0 | 0 | 0 | 100 | 100 | 0 | 0 | 0 | 0 | 0 |
| 4 | Ground-feeders | 0 | 0 | 0 | 50 | 50 | 43 | 0 | 0 | 7 | 50 |
| 5 | Hawkers | 0 | 50 | 0 | 0 | 50 | 0 | 50 | 0 | 0 | 50 |
| 6 | Predators | 0 | 0 | 0 | 100 | 100 | 0 | 0 | 0 | 0 | 0 |
| 7 | Vegivores | 0 | 33 | 0 | 33 | 66 | 0 | 0 | 33 | 0 | 33 |
| 8 | Overall | 0 | 12 | 0 | 55 | 67 | 21 | 3 | 6 | 3 | 33 |

**Panel 2: Agricultural**

| Row | Feeding guild | Positive Interaction Cases | | | | | Negative Interaction Cases | | | | | |
| --- | --- | --- | --- | --- | --- | --- | --- | --- | --- | --- | --- | --- |
|  |  | A | B | C | D | Total | E | F | G | H | Total |  |
| 1 | Frugivores | 0 | 0 | 0 | 40 | 40 | 60 | 0 | 0 | 0 | 60 |  |
| 2 | Gleaners | 0 | 0 | 17 | 66 | 83 | 17 | 0 | 0 | 0 | 17 |  |
| 3 | Granivores | 0 | 8 | 0 | 53 | 61 | 0 | 8 | 23 | 8 | 39 |  |
| 4 | Ground-feeders | 0 | 9 | 0 | 45 | 54 | 32 | 0 | 0 | 14 | 46 |  |
| 5 | Hawkers | 0 | 0 | 0 | 50 | 50 | 50 | 0 | 0 | 0 | 50 |  |
| 6 | Predators | 0 | 0 | 0 | 67 | 67 | 33 | 0 | 0 | 0 | 33 |  |
| 7 | Vegivores | 0 | 0 | 0 | 20 | 20 | 80 | 0 | 0 | 0 | 80 |  |
| 8 | Overall | 0 | 5 | 3 | 50 | 58 | 29 | 2 | 5 | 6 | 42 |  |
